# Vanzacaftor–tezacaftor–deutivacaftor for children aged 6–11 years with cystic fibrosis (RIDGELINE Trial VX21-121-105): an analysis from a single-arm, phase 3 trial

**DOI:** 10.1016/S2213-2600(24)00407-7

**Published:** 2025-01-02

**Authors:** Jordana E Hoppe, Ajay S Kasi, Jessica E Pittman, Renee Jensen, Lena P Thia, Philip Robinson, Pornchai Tirakitsoontorn, Bonnie Ramsey, Marcus A Mall, Jennifer L Taylor-Cousar, Edward F McKone, Elizabeth Tullis, Danieli B Salinas, Jiaqiang Zhu, Yih-Chieh Chen, Violeta Rodriguez-Romero, Patrick R Sosnay, Gwyneth Davies

**Affiliations:** University of Colorado School of Medicine and Children’s Hospital Colorado, Aurora, CO, USA; Emory University School of Medicine and Children’s Healthcare of Atlanta, Atlanta, GA, USA; Washington University School of Medicine, St Louis, MO, USA; The Hospital for Sick Children, Toronto, ON, Canada; Noah’s Ark Children’s Hospital for Wales, Cardiff, UK; The Royal Children’s Hospital, Murdoch Children’s Institute Melbourne, VIC, Australia; Department of Pediatrics, University of Melbourne, Melbourne, VIC, Australia; Children’s Hospital of Orange County, Irvine, CA, USA; University of California, Irvine, CA, USA; Seattle Children’s Hospital, University of Washington, Seattle, WA, USA; Department of Pediatric Respiratory Medicine, Immunology and Critical Care Medicine, Charité - Universitätsmedizin Berlin, Berlin, Germany; German Center for Child and Adolescent Health (DZKJ), partner site Berlin, Berlin, Germany; German Center for Lung Research (DZL), associated partner site Berlin, Berlin, Germany; National Jewish Health, Denver, CO, USA; St Vincents University Hospital, University College Dublin, Dublin, Ireland; St Michael’s Hospital, University of Toronto, Toronto, ON, Canada; Vertex Pharmaceuticals, Boston, MA, USA; Vertex Pharmaceuticals, Boston, MA, USA; Vertex Pharmaceuticals, Boston, MA, USA; Vertex Pharmaceuticals, Boston, MA, USA; Vertex Pharmaceuticals, Boston, MA, USA; Population Policy and Practice Department, UCL Great Ormond Street Institute of Child Health, University College London, London, UK; Great Ormond Street Hospital for Children NHS Foundation Trust, London, UK

## Abstract

**Background:**

In phase 2 trials in people with cystic fibrosis aged 18 years and older, vanzacaftor–tezacaftor–deutivacaftor has been shown to be a safe and effective, once-daily cystic fibrosis transmembrane conductance regulator (CFTR) modulator. Restoring normal CFTR function early in life has the potential to prevent manifestations of cystic fibrosis. We aimed to evaluate the safety, tolerability, efficacy, and pharmacokinetics of vanzacaftor–tezacaftor–deutivacaftor in children with cystic fibrosis aged 6–11 years.

**Methods:**

In this multicentre, single-arm, phase 3 trial (RIDGELINE Trial VX21–121-105), participants were enrolled across 33 clinical sites that care for children with cystic fibrosis in eight countries (Australia, France, Germany, Netherlands, Sweden, Switzerland, the UK, and the USA). Eligible participants were aged 6–11 years with at least one elexacaftor–tezacaftor–ivacaftor-responsive *CFTR* variant, FEV_1_ % predicted of 60% or higher, and stable cystic fibrosis as determined by investigators. Before study treatment, participants were either on stable elexacaftor–tezacaftor–ivacaftor for at least 28 days before screening or received the combination for a 4-week run-in period. Participants then received vanzacaftor–tezacaftor–deutivacaftor (<40 kg bodyweight: vanzacaftor 12 mg, tezacaftor 60 mg, and deutivacaftor 150 mg orally as three fixed-dose combination tablets once daily; ≥40 kg bodyweight: vanzacaftor 20 mg, tezacaftor 100 mg, and deutivacaftor 250 mg orally as two fixed-dose combination tablets once daily (manufactured by Patheon Pharmaceuticals, Cincinnati, OH, USA) from day 1 for 24 weeks. The primary endpoint was safety and tolerability, as measured by adverse events, vital signs, clinical laboratory values, electrocardiograms, and pulse oximetry. Endpoints were analysed in all participants who received at least one dose of vanzacaftor–tezacaftor–deutivacaftor. This trial is registered with ClinicalTrials.gov, NCT05422222, and evaluation of the 6–11-year-old cohort is complete.

**Findings:**

Between Feb 6 and May 18, 2023, 83 children were screened, of whom five were not eligible, and 78 children aged 6–11 years received at least one dose of vanzacaftor–tezacaftor–deutivacaftor. Median age was 9·3 years (IQR 7·6–10·4), 34 (44%) of 78 participants were female, 44 (56%) were male, 71 (91%) were White, one (1%) was Black or African American, and one (1%) was of multiple races. The analysis for these data was completed on Dec 15, 2023. Median exposure of participants to vanzacaftor–tezacaftor–deutivacaftor was 168 days (IQR 166–170). 75 (96%) of 78 participants had adverse events, all of which were mild or moderate; the most common events were generally consistent with cystic fibrosis manifestations, including, cough (36 [46%]), pyrexia (16 [21%]), headache (14 [18%]), infective pulmonary exacerbation of cystic fibrosis (13 [17%]), and oropharyngeal pain (13 [17%]). Serious adverse events occurred in six (8%) participants (two had infective pulmonary exacerbation, one of whom also had failure to thrive; one participant each had adenovirus infection, constipation, pulmonary function test decreased, and cough), and one (1%) participant discontinued due to adverse events of cough and fatigue that were considered possibly related to study drug.

**Interpretation:**

Vanzacaftor–tezacaftor–deutivacaftor was safe and well tolerated and maintained FEV_1_ % predicted from elexacaftor–tezacaftor–ivacaftor baseline with further improved CFTR function. Improvements in CFTR function compared with baseline elexacaftor–tezacaftor–ivacaftor values demonstrate the potential opportunity to restore normal physiology early and prevent development or progression of cystic fibrosis. Nearly all participants had sweat chloride below the diagnostic threshold for cytstic fibrosis (<60 mmol/L) and more than half had normal levels (<30 mmol/L). Additional long-term data in children with cystic fibrosis are being collected in an open-label extension study to demonstrate clinical benefits and safety. These findings will inform health-care providers and people with cystic fibrosis regarding the benefits of early initiation of CFTR modulators.

**Funding:**

Vertex Pharmaceuticals.

## Introduction

Cystic fibrosis is a life-shortening, autosomal recessive disease caused by variants in the cystic fibrosis transmembrane conductance regulator (*CFTR*) gene that affects more than 92 000 individuals globally.^1,2^ There are over 2000 *CFTR* variants, although *F508del* is the most common variant in tested populations with cystic fibrosis to date, and allelic frequency varies across geographical regions. *CFTR* variants can lead to decreased CFTR quantity and function at the cell surface, resulting in an inability to regulate chloride transport in tissues such as those in the lungs, pancreas and other gastrointestinal organs, and sweat glands.^3^ Cystic fibrosis pathophysiology starts in intrauterine life, as suggested by a high prevalence of pancreatic damage, vas deferens occlusion, and meconium ileus in neonates with the disease and by infants having elevated concentrations of chloride in their sweat (characteristic of CFTR dysfunction).^4,5^ Poor nutrition, which is associated with pancreatic insufficiency and increased energy expenditure, has been associated with progressive loss of lung function and increased risk of death.^6,7^ Progressive lung disease might appear early in life, with pulmonary infection, inflammation, and structural lung damage occurring in children as young as 3 months.^8^

CFTR modulators target the underlying cause of cystic fibrosis by improving CFTR function systemically, including in the lungs and sweat glands. Elexacaftor–tezacaftor–ivacaftor, the current standard of care CFTR modulator therapy approved for people aged 2 years and older with cystic fibrosis and at least one eligible variant and who have access, improves lung function (as measured by both FEV_1_ % predicted and lung clearance index [LCI]), respiratory symptoms, and CFTR function (as measured by reduced concentrations of chloride in sweat).^9–13^ Real-world data from people receiving elexacaftor–tezacaftor–ivacaftor showed that this drug combination modifies the disease course, improves survival, and reduces lung transplant rates.^9,10,14–16^ Treatment with CFTR modulators early in life has the potential to prevent manifestations of cystic fibrosis. Although additional data are needed to determine the lifetime clinical benefits of early treatment with CFTR modulators, observational and registry studies have shown that children and adolescents who receive CFTR modulator treatment early in life (eg, at age 6 years) have higher mean FEV_1_ % predicted and lower rates of pulmonary exacerbations, hospitalisations, transplants, and death.^17,18^

The goal of CFTR modulator treatment is to restore normal levels of CFTR function, because people with normal CFTR function generally do not have manifestations of cystic fibrosis disease. Sweat chloride testing is a direct and sensitive measure of CFTR function in the sweat gland and is used for the diagnosis of cystic fibrosis: sweat chloride concentrations of 60 mmol/L or higher are diagnostic for cystic fibrosis, concentrations of 30 mmol/L to less than 60 mmol/L indicate cystic fibrosis is possible with additional evaluation needed, and concentrations below 30 mmol/L are considered normal levels and not consistent with diagnosis of cystic fibrosis according to Cystic Fibrosis Foundation guidelines.^19^ Analysis of data from the US Cystic Fibrosis Foundation Patient Registry found that people with cystic fibrosis with better CFTR function (sweat chloride concentration <60 mmol/L) have better clinical outcomes (including survival, annual rate of FEV_1_ decline, and nutritional status) than do those with worse CFTR function (sweat chloride concentration ≥60 mmol/L).^20^ The association between CFTR function and clinical outcomes has also been seen in pooled clinical trial data of CFTR modulators, where participants aged 12 years or older who had sweat chloride concentrations of less than 60 mmol/L or less than 30 mmol/L while on CFTR modulator therapy had improved clinical outcomes (including lung function, quality of life, nutritional parameters, and pulmonary exacerbation rate) compared with participants with concentrations of 60 mmol/L or higher (unpublished data).^21^ These data support that restoring CFTR function below the diagnostic threshold of cystic fibrosis (sweat chloride concentration <60 mmol/L) and to normal levels (sweat chloride concentration <30 mmol/L) has the potential for clinical benefit, particularly when started early in life.

Vanzacaftor–tezacaftor–deutivacaftor is a once daily, next-generation CFTR modulator regimen developed to further restore CFTR function in people with cystic fibrosis.^22^ The clinical development plan included two randomised controlled phase 3 trials in participants aged 12 years and older (Trials VX20–121-102 and VX20–121-103)^23^ and an open-label phase 3 trial of vanzacaftor–tezacaftor–deutivacaftor in children younger than 12 years with cystic fibrosis (Trial VX21–121-105). Taken together, these trials represent the most robust initial phase 3 programme for CFTR modulators in terms of duration, number of participants, eligible genotypes, geography, and age to date. Trials VX20–121-102 and VX20–121-103 showed that vanzacaftor–tezacaftor–deutivacaftor was non-inferior in FEV_1_ % predicted improvements and superior in restoration of CFTR function compared with elexacaftor–tezacaftor–ivacaftor, and was generally safe and well tolerated.^23^ Consistent with ICH E11 guidance on clinical investigation of medicinal products in the paediatric population, and previous CFTR modulator development programmes, efficacy in children younger than 12 years can be extrapolated from phase 3 trials in participants aged 18 years or older on the basis of supportive efficacy, safety, and exposure results.^24^ Here we describe safety, pharmacokinetic, and efficacy results from Trial VX21–121-105 in children with cystic fibrosis aged 6–11 years who have been treated with vanzacaftor–tezacaftor–deutivacaftor for 24 weeks.

## Methods

### Study design and participants

In this multicentre, single-arm, phase 3 trial (RIDGELINE Trial VX21–121-105), vanzacaftor–tezacaftor–deutivacaftor is being assessed in children aged 1–11 years with cystic fibrosis in three separate descending cohorts according to age (6–11 years, 2–5 years, and 1 year to <2 years). Herein, we report the investigation of vanzacaftor–tezacaftor–deutivacaftor in participants aged 6–11 years. In the age 6–11-years cohort, we evaluated the pharmacokinetics, safety, and tolerability for 22 days (part A) followed by the evaluation of safety, pharmacokinetics, and efficacy for 24 weeks (part B); here, we report data for part B of the trial (data for part A are to be reported elsewhere). Children aged 6–11 years with cystic fibrosis were recruited at 33 clinical sites that care for children with cystic fibrosis in Australia, France, Germany, Netherlands, Sweden, Switzerland, the UK, and the USA (appendix p 16).

Eligible children aged 6–11 years had a confirmed diagnosis of cystic fibrosis with at least one elexacaftor–tezacaftor–ivacaftor-responsive *CFTR* variant, FEV_1_ % predicted of 60% or higher, and stable cystic fibrosis as judged by the recruiting investigators. All participants and their parents or caregivers agreed for the participant to continue their usual cystic fibrosis medication regimens throughout the trial period. Full eligibility criteria are in the appendix (pp 5–8). Data on sex were collected from medical records, and ethnicity and race were collected on the basis of the self-identification provided by the parent or caregiver, as allowed by local regulations.

An independent review board or ethics committee for each site approved the trial protocol and informed consent forms (Central IRB was Advarra, approval number MOD01379147; additional information is provided in the appendix [p 17]). The parents or legal guardian of each enrolled child provided written informed consent and the participant provided signed assent. This trial is registered with ClinicalTrials.gov, NCT05422222, and trial protocol is available online.

### Procedures

**Figure 1:**
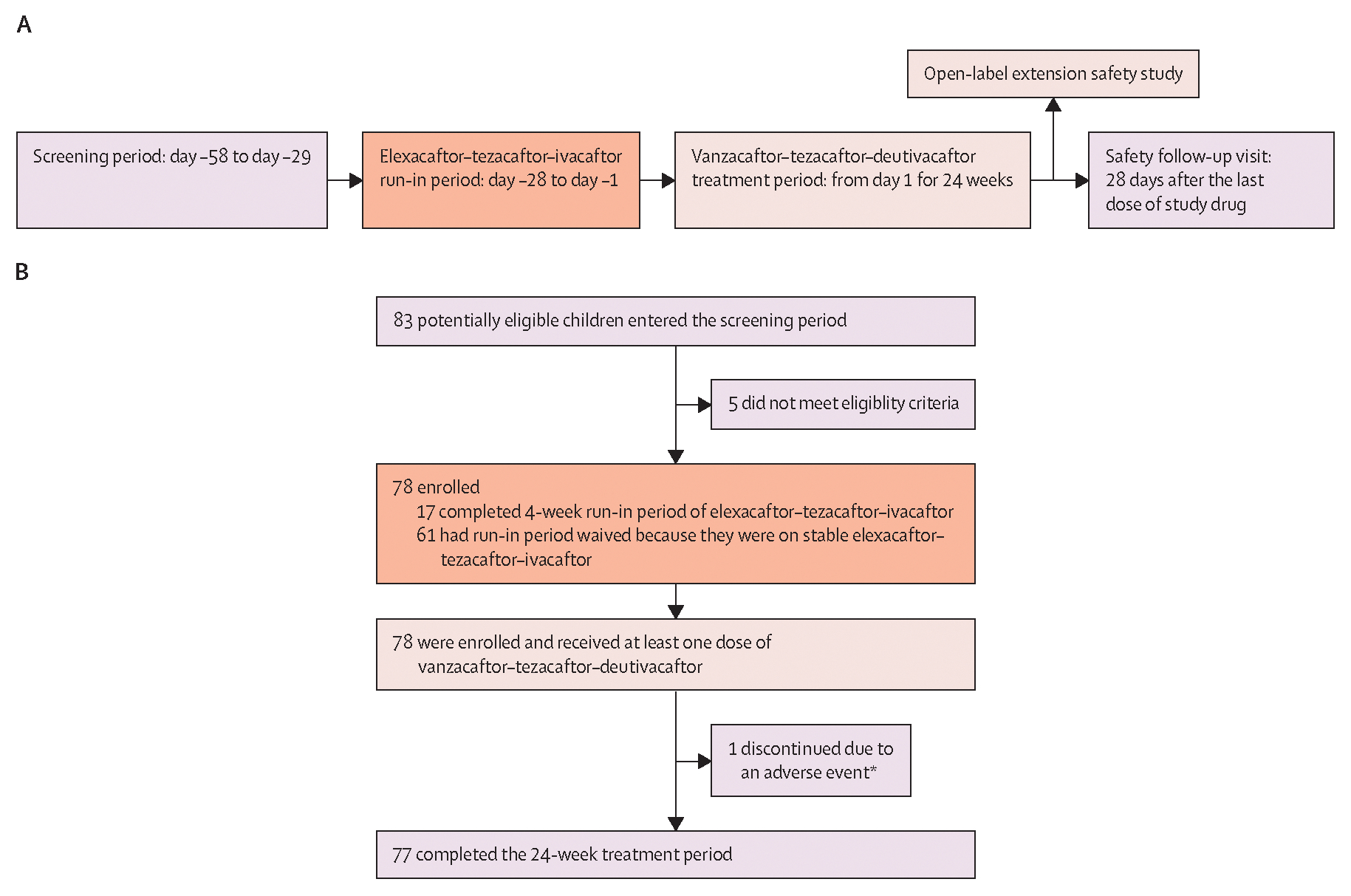
Trial design (A) and trial profile (B) *One child discontinued treatment due to adverse events of cough and fatigue, which were considered possibly related to study drug; cough and fatigue improved following discontinuation, and the child resumed commercial elexacaftor–tezacaftor–ivacaftor.

Before the vanzacaftor–tezacaftor–deutivacaftor open-label treatment period, all children were either on a stable regimen of elexacaftor–tezacaftor–ivacaftor (defined as receiving elexacaftor–tezacaftor–ivacaftor for at least 28 days before the screening visit) or received elexacaftor–tezacaftor–ivacaftor for 4 weeks as part of the study run-in period to establish a stable on-treatment baseline (figure 1A). During the run-in period, participants weighing less than 30 kg on day –28 received elexacaftor 100 mg and tezacaftor 50 mg once a day and ivacaftor 75 mg once every 12 h (as a fixed-dose combination tablet and an ivacaftor tablet); participants weighing 30 kg or more on day –28 received elexacaftor 200 mg and tezacaftor 100 mg once a day, and ivacaftor 150 mg once every 12 h (as a fixed-dose combination tablet and an ivacaftor tablet; manufactured at Vertex Pharmaceuticals, Boston, MA, USA, and Patheon Pharmaceuticals, Cincinnati, OH, USA).

During the vanzacaftor–tezacaftor–deutivacaftor open-label treatment period, children who weighed less than 40 kg on day 1 received vanzacaftor 12 mg, tezacaftor 60 mg, and deutivacaftor 150 mg orally as three fixed-dose combination tablets once daily; children who weighed 40 kg or more on day 1 received vanzacaftor 20 mg, tezacaftor 100 mg, and deutivacaftor 250 mg orally as two fixed-dose combination tablets once daily (manufactured by Patheon Pharmaceuticals, Cincinnati, OH, USA). Participants received study drug for the 24-week treatment period. Details regarding schedules of assessments, follow-up, study drug interruption, stopping criteria, and adverse event categorisation and reporting criteria are in the appendix (pp 8–12, 14). After last dose of study drug, participants had a safety follow-up visit within 28 days or, for participants who completed the trial, had the opportunity to participate in an open-label extension trial in which they continued to receive vanzacaftor–tezacaftor–deutivacaftor (NCT05844449).

### Outcomes

The primary endpoint for part B was safety and tolerability as measured by adverse events, vital signs, clinical laboratory values, electrocardiograms, and pulse oximetry. Pharmacokinetics parameters of vanzacaftor, tezacaftor, and deutivacaftor were a secondary endpoint. Secondary efficacy endpoints were measures of absolute change in sweat chloride concentration; absolute change in FEV_1_ % predicted; absolute change in Cystic Fibrosis Questionnaire-Revised (CFQ-R) respiratory domain score; number of protocol-defined pulmonary exacerbations (defined in the appendix [p 13]) and cystic fibrosis-related hospitalisations through week 24; absolute change in BMI, weight, and height and corresponding Z scores at week 24; the proportion of children with sweat chloride concentrations below 60 mmol/L and below 30 mmol/L through week 24; and drug acceptability assessment using Modified Facial Hedonic Scale. Secondary endpoints which were analysed through week 24, except for pulmonary exacerbation, were estimated by averaging measurements from weeks 16 and 24; for number of pulmonary exacerbations and cystic fibrosis-related hospitalisations, these were summarised over the treatment period. Other endpoints were absolute change in faecal elastase-1 (FE-1) at week 24, absolute change in serum immunoreactive trypsinogen at week 24, absolute change in faecal calprotectin at week 24, and absolute change in lung clearance index_2·5_ (defined as the number of lung turnovers required to reduce the end tidal inert gas concentration to one-fortieth of its starting value) through week 24 (estimated by averaging the values at weeks 12 and 24), and absolute change in fat soluble vitamins from baseline through week 24. Outcomes of drug acceptability assessment using Modified Facial Hedonic Scale and absolute change in fat soluble vitamins from baseline though week 24 were included to inform further clinical development in paediatric cohorts and are not reported here.

### Statistical analysis

No formal power calculation was performed. Approximately 65 children were planned for enrolment, with approximately 55 expected to complete the trial. With approximately 55 children completing the trial, there was a more than 90% chance of observing an adverse event in at least one participant if the true incidence rate was 5%, and a more than 95% chance of observing an adverse event in at least one participant if the true incidence rate was 10%. Probabilities were calculated by assuming a binomial distribution for the number of adverse events. Selection of these true incidence rates was consistent with previous pivotal phase 3 studies of CFTR modulators in this age range.^11^

Safety and pharmacokinetics analyses included children who received at least one dose of vanzacaftor–tezacaftor–deutivacaftor in the treatment period. Efficacy analyses included all children who were enrolled, carried the intended *CFTR* genotype, and received at least one dose of study treatment during the treatment period. Descriptive analyses were done for the safety data; no statistical hypothesis testing was performed. For continuous variables, number of participants, mean (SD), and median (IQR) were calculated. Count and percentage were calculated for categorical variables. Absolute change from baseline in sweat chloride concentration was analysed using a mixed-effects model for repeated measures (MMRM) approach. The MMRM was used to estimate the within-group mean absolute change in sweat chloride concentration from baseline through week 24 (estimated by averaging weeks 16 and 24). The model included absolute change from baseline in sweat chloride concentration at day 15, week 4, week 16, and week 24 as the dependent variable, with fixed categorical effects for genotype group (*F508del*-minimal function, *F508del*-*F508del*, and other) and visit, and baseline sweat chloride concentration as a continuous covariate. The model was estimated using restricted maximum likelihood. Denominator degrees of freedom for the F-test for fixed effects were estimated using the Kenward–Roger approximation.^25^ An unstructured covariance structure was used to model the within-participant errors. The estimated mean change from baseline through week 24 along with the two-sided 95% CI was provided, along with the estimated mean change and its SE at each post-baseline visit. Absolute change from baseline in FEV_1_ % predicted, CFQ-R respiratory domain score, BMI, BMI Z score, weight, weight Z score, height, height Z score, and LCI_2.5_ were analysed using an MMRM similar to that described earlier.

The number and proportion of participants with sweat chloride concentrations below 60 mmol/L and below 30 mmol/L was summarised descriptively. The two-sided 95% CI based on the Clopper–Pearson method was presented. The number of protocol-defined pulmonary exacerbations was analysed descriptively. Absolute change from baseline in FE-1, shift from baseline category (<200 mg/kg *vs* ≥200 mg/kg) in FE-1, serum immunoreactive trypsinogen, and faecal calprotectin were analysed using descriptive statistics.

Post-hoc analyses by genotype subgroup were performed in a similar manner to the main efficacy analysis for absolute change from baseline in FEV_1_ % predicted and sweat chloride concentrations and sweat chloride response (<60 mmol/L and <30 mmol/L). A post-hoc descriptive analysis was also performed to assess the absolute change from baseline in LCI_2·5_ by baseline values (≤7·5 and >7·5). For the rate of protocol-defined pulmonary exacerbations, the 95% CI was obtained assuming a negative binomial distribution as a post-hoc analysis.

Prespecified population pharmacokinetic modelling was conducted using non-linear mixed-effects modelling in NONMEM versions 7.4.4 and 7.5.1 to allow comparison with adult populations from Trials VX20–121-102 and VX20–121-103.^23^

SAS version 9.4 or higher was used to generate all statistical outputs. Safety was monitored by an independent data monitoring committee.

### Role of the funding source

The trial sponsor (Vertex Pharmaceuticals Incorporated) had a role in the study design, data analysis, data interpretation, and writing of the report. The sponsor had no role in data collection.

## Results

Between Feb 6 and May 18, 2023, 83 children were screened, of whom five were not eligible; 78 children were enrolled. Of the 78 enrolled children, 17 completed the elexacaftor–tezacaftor–ivacaftor run-in period before the treatment period and 61 had the run-in period waived because they were on stable elexacaftor–tezacaftor–ivacaftor before screening. 78 participants received at least one dose of vanzacaftor–tezacaftor–deutivacaftor in the treatment period (figure 1B). One participant had adverse events that led to vanzacaftor–tezacaftor–deutivacaftor discontinuation and all others completed treatment. Median age was 9·3 years (IQR 7·6–10·4), 34 (44%) of 78 participants were female, 44 (56%) were male, 71 (91%) were White, one (1%) was Black or African American, one (1%) was of multiple races, and five (6%) did not have data on race collected (table 1; additional baseline characteristics are in the appendix [p 18]). The analysis for these data was completed on Dec 15, 2023. Median exposure of participants to vanzacaftor–tezacaftor–deutivacaftor was 168 days (IQR 166–170). After completion of the trial, 75 of 78 participants enrolled in an open-label extenstion study assessing long-term safety and efficacy.

75 (96%) of 78 participants had at least one adverse event, all of which were mild or moderate in severity (table 2). The most common adverse events and serious adverse events were mostly consistent with common manifestations of cystic fibrosis, including, cough (36 [46%]), pyrexia (16 [21%]), headache (14 [18%]), infective pulmonary exacerbation of cystic fibrosis (13 [17%]), and oropharyngeal pain (13 [17%]; table 2). Serious adverse events occurred in six (8%) participants (two had infective pulmonary exacerbation of cystic fibrosis, one of whom also had failure to thrive; and one participant each had adenovirus infection, constipation, pulmonary function test decreased, and cough). One participant discontinued vanzacaftor–tezacaftor–deutivacaftor due to adverse events of cough and fatigue which were not associated with changes in FEV_1_ % predicted, started on day 30, and were considered possibly related to vanzacaftor–tezacaftor–deutivacaftor. The participant was treated for these symptoms, which resolved after discontinuation of study treatment. This participant subsequently resumed commercial elexacaftor–tezacaftor–ivacaftor.

Safety data for known adverse drug reactions with elexacaftor–tezacaftor–ivacaftor (eg, increased alanine aminotransferase or aspartate aminotransferase and rash), possible risks associated with elexacaftor–tezacaftor–ivacaftor (eg, cataracts), and events that are common in individuals with cystic fibrosis (eg, neuropsychiatric events) were additionally reviewed. Three (4%) participants had alanine aminotransferase or aspartate aminotransferase concentrations that were more than three times the upper limit of normal (ULN), one (1%) participant had concentrations more than five times the ULN, and no participants had concentrations more than eight times the ULN (appendix p 18). No participants had alanine aminotransferase or aspartate aminotransferase concentrations more than three times the ULN concurrently with a newly occurring elevation in total bilirubin more than two times the ULN. There were no serious adverse events, treatment interruptions, or treatment discontinuations related to elevated aminotransferase events. Four (5%) participants had at least one rash event; all of which were mild in severity and did not lead to treatment discontinuation or treatment interruption (appendix p 19). No serious rash events occurred. Median time-to-onset of the first rash event was 9·0 days (IQR 5–11). Four (5%) participants had a neuropsychiatric event, all of which were mild in severity (appendix p 19). No serious neuropsychiatric events occurred, and none led to treatment discontinuation or treatment interruption. There were two events of insomnia (occurring in one participant each that resolved in 1 day and 22 days). One (1%) participant, with a medical history of esotropia, amblyopia, and hyperopia and a screening ophthalmological examination that was deemed normal, had an adverse event of a right posterior cortical cataract on day 170 that was mild in severity, not considered to be visually significant, and not associated with any symptoms; there were no changes to study drug treatment and the participant completed the trial. This participant did not have any known risk factors for cataracts. No other clinically relevant events or patterns were observed in other clinical or laboratory assessments. Mean systolic blood pressure and diastolic blood pressure remained in the normal range during the 24-week treatment period (appendix p 20).

Population pharmacokinetics analyses indicated that exposures of vanzacaftor, tezacaftor, active metabolite 1 (M1) of tezacaftor, and deutivacaftor for children aged 6–11 years were within the exposure range shown to be safe and efficacious in people with cystic fibrosis aged 18 years or older. The steady-state exposures of vanzacaftor, tezacaftor, M1-tezacaftor, and deutivacaftor in cystic fibrosis participants aged 6–11 years, 12–17 years, and aged 18 years or older are compared graphically in the appendix (p 15).

**Figure 2:**
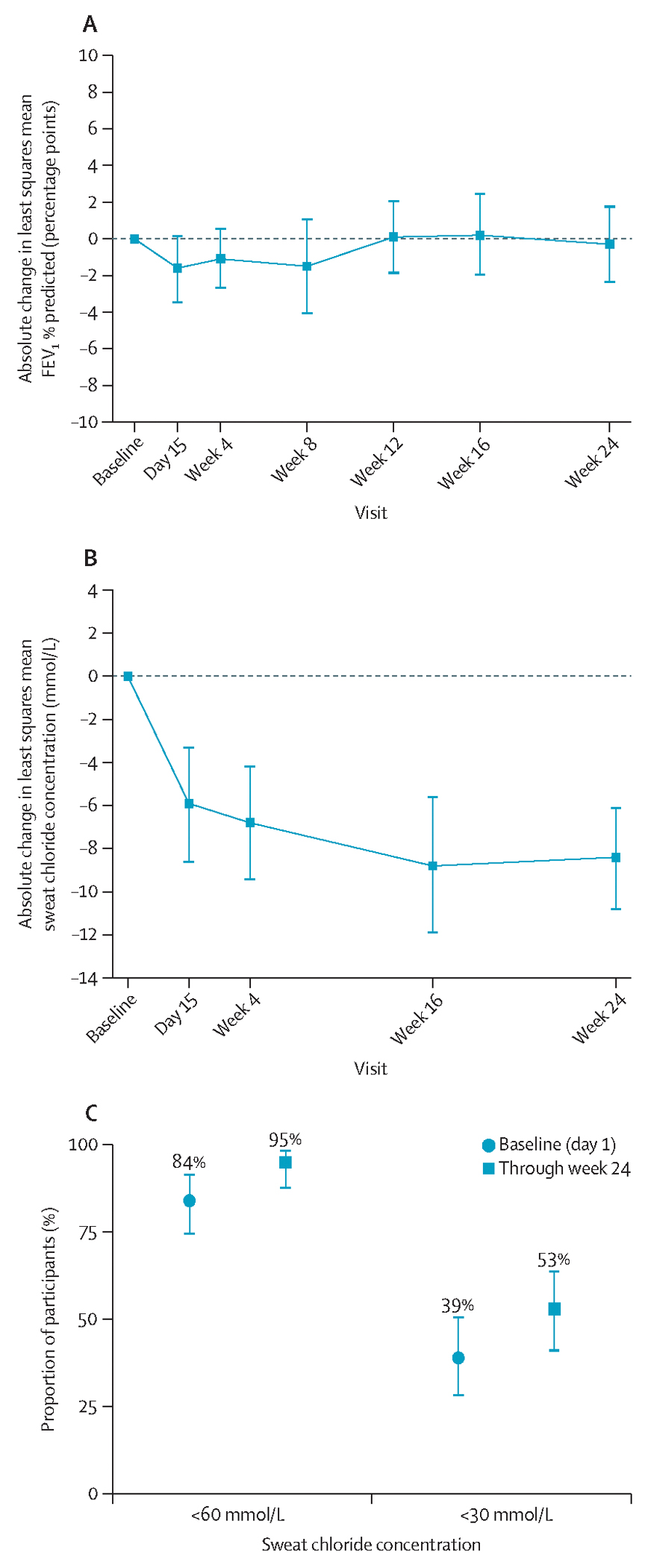
Absolute least squares mean change from baseline in FEV_1_ % predicted (A) and sweat chloride concentration (B), and the proportion of participants with sweat chloride concentrations <60 mmol/L and <30 mmol/L (C) Error bars show 95% CIs.

At baseline (pre-dose day 1), mean FEV_1_ % predicted was within the normal range (99·7 percentage points [SD 15·1]) and mean sweat chloride concentration was 40·4 mmol/L (SD 20·9), both of which reflect the efficacy of elexacaftor–tezacaftor–ivacaftor (table 3). Participants maintained normal baseline FEV_1_ % predicted (least squares mean absolute change from baseline through week 24 was 0·0 percentage points [95% CI −2·0 to 1·9]; table 3, figure 2A; appendix p 24). Least squares mean absolute change from baseline through week 24 in sweat chloride concentrations was −8·6 mmol/L (95% CI −11·0 to −6·3; table 3, figure 2B; appendix p 22), with 94·9% (95% CI 87·4 to 98·6) of participants having sweat chloride concentrations below 60 mmol/L and 52·6% (40·9 to 64·0) having sweat chloride concentrations below 30 mmol/L through week 24, compared with 84·4% and 39·0%, respectively, at baseline (table 3, figure 2C). Results from post-hoc genotype subgroup analyses show stable FEV_1_ % predicted and improved CFTR function in all genotype categories (appendix pp 23, 25–26). Vanzacaftor–tezacaftor–deutivacaftor treatment led to improvements in CFQ-R respiratory domain score, with a least squares mean absolute change of 3·9 points (95% CI 1·5 to 6·3) from baseline through week 24 (minimum clinically important difference is 4 points).^26^ The proportions of missing data for each endpoint over the total data assessment period were 5·1% for sweat chloride, 7·7% for FEV_1_ % predicted, and 3·4% for CFQ-R respiratory domain score. BMI, BMI-for-age Z score, weight, weight-for-age Z score, height, and height-for-age Z score were similar at baseline and at week 24 (table 3; appendix p 21). Increases in weight and height were observed at week 24, consistent with expected growth in this population. Six (8%) participants each had one protocol-defined pulmonary exacerbation, with an annualised event rate of 0·15 (95% CI 0·07 to 0·34) events per year (table 3). There were two planned and three unplanned cystic fibrosis-related hospitalisations, with event rates of 0·05 events per year and 0·08 events per year, respectively.

53 (68%) of 78 participants had a baseline FE-1 value and 45 of these participants also had an FE-1 value at week 24. Mean baseline FE-1 concentration was 133·9 mg/kg (SD 188·6) and the mean absolute change at week 24 was 19·5 mg/kg (95% CI 3·3 to 35·8). Prespecified analysis of FE-1 values showed that, among 41 (77%) of 53 participants with baseline FE-1 concentration below 200 mg/kg (ie, below the threshold for pancreatic sufficiency), two (5%) had an FE-1 concentration of 200 mg/kg or higher at week 24. 23 (43%) of 53 participants with baseline data had an FE-1 concentration of less than 15 mg/kg (definitive pancreatic insufficiency and below the detectable limit of 15 mg/kg) at baseline, and three (13%) of 23 had detectable FE-1 concentrations at week 24. Mean absolute change from baseline at week 24 in serum immunoreactive trypsinogen was −51·5 μg/L (95% CI −120**·**0 to 16·9), and in faecal calprotectin was −31·0 mg/kg (95% CI −69·9 to 7·9). Baseline LCI_2·5_ was 6·63 (SD 0·74) and the least squares mean absolute change through week 24 was −0·08 (–0·18 to 0·02; table 3). A post-hoc analysis showed that participants who had normal-range baseline LCI_2·5_ (≤7·5; n=62) had a mean absolute change from baseline at week 24 of −0·06 (SD 0·48), whereas participants with a baseline LCI_2·5_ outside of the normal range (>7·5; n=10) had a mean absolute change from baseline at week 24 of −0·82 (SD 0·47; appendix p 22). The proportion of missing data for these endpoints over the total data assessment period was 14·1% for LCI_2·5_, 22·1% for FE-1, 21·5% for faecal calprotectin, and 46·9% for immunoreactive trypsinogen.

## Discussion

In this 24-week, open-label, phase 3 trial of vanzacaftor–tezacaftor–deutivacaftor in participants aged 6–11 years with cystic fibrosis who were on stable elexacaftor–tezacaftor–ivacaftor at baseline, treatment was safe and well tolerated. Baseline FEV_1_ % predicted while on stable elexacaftor–tezacaftor–ivacaftor was in the normal range and was maintained after vanzacaftor–tezacaftor–deutivacaftor treatment. Treatment with vanzacaftor–tezacaftor–deutivacaftor resulted in lower sweat chloride concentration through week 24 from the baseline value that was established on elexacaftor–tezacaftor–ivacaftor, with nearly all participants having sweat chloride concentrations below 60 mmol/L and more than half having concentrations below 30 mmol/L through week 24. These thresholds are thought to be clinically significant because health-care providers use them in their diagnosis of cystic fibrosis and data from both natural history and pooled clinical trials suggest that people with cystic fibrosis with sweat chloride concentrations of less than 60 mmol/L and 30 mmol/L have better clinical outcomes than do those with cystic fibrosis and sweat chloride concentration of 60 mmol/L or higher;^20,21^ however, these sweat chloride thresholds have not yet been used prospectively to evaluate response of CFTR modulator therapy.

The safety profile of vanzacaftor–tezacaftor–deutivacaftor is well characterised in this trial and similar to that seen in phase 3 trials in participants aged 12 years and older.^23^ Few serious adverse events and discontinuations occurred. Additional adverse events seen with other CFTR modulators included increased aminotransferases, rash, and cataracts: these events were not serious and none led to drug interruption or discontinuation. In the context of all participants entering the trial on stable elexacaftor–tezacaftor–ivacaftor treatment, the frequency and extent of aminotransferase and rash events in this trial were similar or lower than those seen in participants in the same age group receiving elexacaftor–tezacaftor–ivacaftor in previous clinical trials.^11,12^ Neuropsychiatric events, which are common in people with cystic fibrosis, were not serious, with no events leading to drug interruption or discontinuation, and were consistent with the background rate of these events in people with cystic fibrosis not receiving CFTR modulator treatment, and in people with cystic fibrosis in this age group.^27–31^

Highly effective CFTR modulator regimens capable of restoring CFTR function to the normal range are a major advancement for the treatment of cystic fibrosis. Because the mean baseline lung function (measured by FEV_1_ % predicted) was within the normal range, we were less likely to detect additional improvements by spirometry in this clinical trial. Indeed, normal baseline FEV_1_ % predicted established on elexacaftor–tezacaftor–ivacaftor was maintained with vanzacaftor–tezacaftor–deutivacaftor treatment, which is clinically meaningful because cystic fibrosis is a disease characterised by progressive loss of lung function. Treatment with vanzacaftor–tezacaftor–deutivacaftor led to further decreases in mean sweat chloride concentration and an increase in the proportion of participants with sweat chloride concentrations below 60 mmol/L and 30 mmol/L compared with baseline established on elexacaftor–tezacaftor–ivacaftor, consistent with results from the parallel phase 3 trials in participants aged 12 years and older, which showed that vanzacaftor–tezacaftor–deutivacaftor further restored CFTR function compared with elexacaftor–tezacaftor–ivacaftor.^23^ These findings are especially important because early initiation of CFTR modulator therapy and maintenance of normal levels of CFTR function could be crucial in preventing cystic fibrosis disease.

Notably, numerical improvements in other efficacy measures were observed with vanzacaftor–tezacaftor–deutivacaftor treatment relative to baseline on stable elexacaftor–tezacaftor–ivacaftor treatment. LCI has been described as a more sensitive measure of lung function than FEV_1_ because it reflects the level of ventilation inhomogeneity in the lungs.^32^ Although mean baseline LCI was within the normal range of children without cystic fibrosis, a post-hoc analysis showed that participants who had baseline LCI outside this normal range improved to within the normal range after 24 weeks of treatment. Although the sample size for this post-hoc analysis was small and there was no control group for comparison, this result potentially suggests that vanzacaftor–tezacaftor–deutivacaftor could improve subclinical lung disease that develops early in life in children with cystic fibrosis. Additionally, even with the high baseline CFQ-R respiratory domain score established on elexacaftor–tezacaftor–ivacaftor, numerical improvement was observed over 24 weeks of vanzacaftor–tezacaftor–deutivacaftor treatment. This finding suggests that, similar to lung function, additional benefits in quality-of-life parameters might be possible with improved CFTR function with vanzacaftor–tezacaftor–deutivacaftor treatment. Changes in baseline FE-1 at week 24 showed improvement in this biomarker of pancreatic function, including a small increase in the number of participants who had FE-1 concentrations above the threshold for pancreatic sufficiency and above the detectable limit in post-hoc analysis. Although the ability to fully restore pancreatic exocrine function in this age group is probably limited, these results reinforce that early restoration of CFTR function might be crucial to prevent or at least improve pancreatic exocrine function.

A limitation of this trial is the open-label design, which precludes direct comparisons of the safety and efficacy of vanzacaftor–tezacaftor–deutivacaftor to either placebo or elexacaftor–tezacaftor–ivacaftor. Similarly, because the primary objective of the trial was safety and tolerability, the trial was not designed to demonstrate statistically significant changes in the efficacy endpoints. However, consistent with the principles of ICH E11 (which provides guidance for clinical investigation of medicinal products in the paediatric population) and previous CFTR modulator programmes, extrapolation of efficacy from adults to a younger population can be achieved by showing similar exposures of drug concentrations and similar safety profiles between adults and children.^24^ Given that we did not have a control group in this trial, the possibility of regression to the mean cannot be ruled out when evaluating efficacy results, but this phenomenon is unlikely because baseline efficacy measures on elexacaftor–tezacaftor–ivacaftor were either maintained or improved over time.

Another limitation of the analysis is that enrolled participants were on stable elexacaftor–tezacaftor–ivacaftor (eg, able to tolerate elexacaftor–tezacaftor–ivacaftor); therefore, the efficacy and safety of vanzacaftor–tezacaftor–deutivacaftor in children who do not tolerate elexacaftor–tezacaftor–ivacaftor remains unstudied. We also recognise the potential for selection bias due to missing data for some outcomes. Overall, the proportion of participants with missing data for key efficacy measures was low. For other endpoints, such as FE-1 and faecal calprotectin, the proportion of missing data was more likely to be higher because of challenges in assay and sample collection (eg, participant not being able to produce stool within the 24 h collection period and difficulties in sample collection before the clinic visit)^33^ rather than because of treatment effect. We also note that additional long-term data in participants with cystic fibrosis are being collected in the open-label extension study of this trial to demonstrate the clinical benefits and safety of even greater CFTR function restoration. Finally, we acknowledge that further efforts to improve recruitment of study participants who identify in minority ethnic groups are important for future cystic fibrosis trials so that the study population and clinical trial data more inclusively reflect the expanse of social factors that affect health outcomes of the entire population with cystic fibrosis.

This phase 3 trial of participants aged 6–11 years with cystic fibrosis showed that treatment with vanzacaftor–tezacaftor–deutivacaftor, a next-generation once-daily CFTR modulator regimen, was generally safe and well tolerated and resulted in further restoration of CFTR function from baseline treatment with elexacaftor–tezacaftor–ivacaftor. Additionally, once-daily treatment has the potential for reduced treatment burden and improved adherence, which might lead to better clinical outcomes in people with cystic fibrosis.^34^ Together, Trial VX21–121-105 and Trials VX20–121-102 and VX20–121-103 (in participants aged ≥12 years)^23^ provide the most comprehensive initial phase 3 programme to date for a CFTR modulator regimen to assess safety and efficacy and demonstrate the clinical benefit of vanzacaftor–tezacaftor–deutivacaftor for eligible people with cystic fibrosis.

## Supplementary Material

1

## Figures and Tables

**Table 1:** Baseline demographic and clinical characteristics

	Participants (N=78)

Sex	
Female	34 (44%)
Male	44 (56%)
Age, years	9·3 (7·6–10·4)
Race	
White	71 (91%)
Black or African American	1 (1%)
Not collected per local regulations	5 (6%)
Multiple races	1 (1%)
Ethnicity	
Hispanic or Latino	9 (12%)
Not Hispanic or Latino	62 (79%)
Not collected per local regulations	7 (9%)
Geographical region	
North America	47 (60%)
Europe	23 (29%)
Australia	8 (10%)
FEV_1_ % predicted, percentage points	
Mean	99·7 (15·1)
Median	100·5 (92·1–108·8)
Sweat chloride concentration, mmol/L	
Mean	40·4 (20·9)
Median	39·0 (24·5–50·0)
<60	65 (83%)
<30	30 (38%)
CFQ-R respiratory domain score	
Mean	84·8 (16·1)
Median	91·7 (83·3–91·7)
BMI, kg/m^2^	
Mean	16·8 (2·1)
Median	16·3 (15·2–17·9)
Genotype group	
Heterozygous *F508del*-minimal function variant	24 (31%)
Homozygous *F508del-F508del*	37 (47%)
Heterozygous *F508del*-gating variant	3 (4%)
Heterozygous *F508del*-residual function variant	1 (1%)
Heterozygous *F508del*-other variant	2 (3%)
Heterozygous other triple combination responsive (non-*F508del*) variant-any variant	11 (14%)
Previous CFTR modulator use	
Any	66 (85%)
Elexacaftor–tezacaftor–ivacaftor	62 (79%)
Tezacaftor–ivacaftor	0
Lumacaftor–ivacaftor	2 (3%)
Ivacaftor	2 (3%)

Data are n (%), mean (SD), or median (IQR). Percentages may not add up to 100 due to rounding. CFQ-R=Cystic Fibrosis Questionnaire – Revised. CFTR=cystic fibrosis transmembrane conductance regulator.

**Table 2:** Adverse events during the treatment period

	Participants (N=78)

Any adverse event	75 (96%)
Maximum severity of adverse event	
Mild	39 (50%)
Moderate	36 (46%)
Severe	0
Life-threatening	0
Adverse events leading to discontinuation of trial regimen	1 (1%)
Adverse events leading to interruption of trial regimen	1 (1%)
Serious adverse events	6 (8%)
Adverse events leading to death	0
Adverse events that occurred in ≥10% participants	
Cough	36 (46%)
Pyrexia	16 (21%)
Headache	14 (18%)
Infective pulmonary exacerbation of cystic fibrosis	13 (17%)
Oropharyngeal pain	13 (17%)
Abdominal pain	9 (12%)
Nasal congestion	9 (12%)
Rhinorrhoea	9 (12%)
Vomiting	8 (10%)
Participants with serious adverse events	
Any	6 (8%)
Infective pulmonary exacerbation of cystic fibrosis	2 (3%)
Adenovirus infection	1 (1%)
Cough	1 (1%)
Failure to thrive	1 (1%)
Pulmonary function test decreased	1 (1%)
Constipation*	1 (1%)

Data are n (%).

* Participant had a history of constipation; the serious adverse event of constipation was considered possibly related to study drug the participant was treated with macrogol and the event resolved with continued study drug dosing.

**Table 3:** Secondary endpoints and select other endpoints

	Participants (N=78)

**Absolute change in FEV_1_ % predicted from baseline through week 24 (percentage points)**	
Baseline, mean (SD; n=77)	99·7 (15·1)
Absolute change, least squares mean (95% CI; n=74)	0·0 (−2·0 to 1·9)
**Absolute change in sweat chloride concentration from baseline through week 24 (mmol/L)**	
Baseline, mean (SD; n=77)	40·4 (20·9)
Absolute change, least squares mean (95% CI; n=77)	−8·6 (−11·0 to −6·3)
**Proportion of participants with sweat chloride <60 mmol/L through week 24**	
Baseline proportion	65/77 (84·4%)
Proportion through week 24*	74/78 (94·9%; 87·4% to 98·6%)
**Proportion of participants with sweat chloride <30 mmol/L through week 24**	
Baseline proportion	30/77 (39·0%)
Proportion through week 24*	41/78 (52·6%; 40·9% to 64·0%)
**Absolute change in CFQ-R respiratory domain score from baseline through week 24 (points)**	
Baseline, mean (SD; n=75)	84·8 (16·1)
Absolute change, least squares mean (95% CI; n=75)	3·9 (1·5 to 6·3)
**Absolute change in BMI from baseline at week 24, kg/m^2^**	
Baseline, mean (SD; n=78)	16·83 (2·13)
Absolute change, least squares mean (95% CI; n=78)	0·22 (0·05 to 0·38)
**Absolute change in BMI Z score from baseline at week 24**	
Baseline, mean (SD: n=78)	0·07 (0·87)
Absolute change, least squares mean (95% CI; n=78)	−0·05 (−0·12 to 0·02)
**Absolute change in LCI_2.5_ from baseline through week 24**	
Baseline, mean (SD; n=72)	6·63 (0·74)
Absolute change, least squares mean (95% CI; n=67)	−0·08 (−0·18 to 0·02)
**Pulmonary exacerbations**	
Number of participants with events, n (%)	6 (7·7%)
Number of events	6
Observed event rate per year (95% CI)	0·15 (0·07 to 0·34)
**Cystic fibrosis-related hospitalisations**	
Planned hospitalisations	
Number of participants with events, n (%)	2 (3%)
Number of events	2
Observed event rate per year	0·05
Unplanned hospitalisations	
Number of participants with events, n (%)	3 (4%)
Number of events	3
Observed event rate per year	0·08

Data for endpoints reporting proportions of participants are n/N1 (%) or n/N1 (% 95% CI), where n is the size of the subsample, and N1 is the number of participants with non-missing data at week 16 or week 24. Baseline was defined as the pre-dose day 1 value. CFQ-R=Cystic Fibrosis Questionnaire–Revised. LCI_2.5_=number of lung turnovers required to reduce the end tidal inert gas concentration to 1/40th of its starting value.

* Obtained by averaging data from week 16 and week 24 visits.

## Data Availability

Vertex Pharmaceuticals is committed to advancing medical science and improving patient health, including the responsible sharing of de-identified clinical trial data with qualified researchers. Proposals for the use of these data will be reviewed by a scientific board. Approvals are at the discretion of Vertex and will be dependent on the nature of the request, the merit of the research proposed, and the intended use of the data. Contact CTDS@vrtx.com if you would like to submit a proposal or need more information.

## Research in context

### Evidence before this study

We searched PubMed for articles published in English from database inception to Nov 20, 2024, using the terms “CFTR modulator”, “clinical trial”, “vanzacaftor”, and “elexacaftor”. Cystic fibrosis is a progressive multi-system disease, with the greatest morbidity affecting the respiratory and gastrointestinal systems. In the absence of curative treatments, the goal is to minimise the effect of disease and prevent, or at least delay, progression. Cystic fibrosis lung disease begins in infancy, underpinning the rationale for starting CFTR modulators—treatments that target the underlying molecular defect in cystic fibrosis—early in childhood and before irreversible pathological changes ensue. For children with cystic fibrosis with an eligible genotype and access to therapy, the CFTR modulator elexacaftor–tezacaftor–ivacaftor has already rapidly transformed the clinical landscape. Although substantial improvement in clinical outcomes including lung function and reduction in pulmonary exacerbations has been achieved with elexacaftor–tezacaftor–ivacaftor, measurements of chloride concentrations in sweat (a sensitive assay for CFTR function) suggest that the opportunity to further improve CFTR function exists.

### Added value of this study

To our knowledge, this trial is the first to evaluate vanzacaftor–tezacaftor–deutivacaftor treatment in children (aged 6–11 years) with cystic fibrosis. With safety and tolerability as primary outcomes, we found that the combination treatment was safe and well tolerated in these paediatric participants after 24 weeks of treatment. In conjunction with the safety data, efficacy data reported here provide evidence that further correction of CFTR function beyond that reached with the current triple combination CFTR modulator (elexacaftor–tezacaftor–ivacaftor) is possible.

### Implications of all the available evidence

In combination with trials VX20–121–102 and VX20–121–103, which assessed this drug combination in participants aged 12 years and older, this paediatric trial provides evidence in support of the efficacy and safety of vanzacaftor–tezacaftor–deutivacaftor compared with baseline elexacaftor-tezacaftor-ivacaftor treatment. Because the prevention or delay of organ system manifestations of cystic fibrosis are a key goal of CFTR modulator development, data from these three phase 3 trials are simultaneously available and have been jointly submitted both for publication and to the regulatory authorities for approval. Vanzacaftor–tezacaftor–deutivacaftor is a new triple combination CFTR modulator treatment that could further advance the goal of restoring normal CFTR function. By initiating such treatments earlier in childhood, there is greater potential to modify progression of cystic fibrosis disease.
