# Supplementary material for: Vanzacaftor–tezacaftor–deutivacaftor for children aged 6–11 years with cystic fibrosis (RIDGELINE Trial VX21-121-105): an analysis from a single-arm, phase 3 trial
Source: Lancet Respir Med. Author manuscript; Available in PMC 2025 May 31. (PMC12126198; doi:10.1016/S2213-2600(24)00407-7)
Supplement: 1 [file NIHMS2081041-supplement-1.pdf]

THE LANCET
Respiratory Medicine

Supplementary appendix

This appendix formed part of the original submission and has been peer reviewed. We post it as supplied by the authors.

Supplement to: Hoppe JE, Kasi AS, Pittman JE, et al. Vanzacaftor–tezacaftor–deutivacaftor for children aged 6–11 years with cystic fibrosis (RIDGELINE Trial VX21-121-105): an analysis from a single-arm, phase 3 trial. *Lancet Respir Med* 2025; published online Jan 2. https://doi.org/10.1016/S2213-2600(24)00407-7.

Appendix to:

Vanzacaftor/Tezacaftor/Deutivacaftor for Children with Cystic Fibrosis: Results From an Open-label Phase 3 Trial

Jordana E. Hoppe MD,[1] Ajay S. Kasi MD,[2] Jessica E. Pittman MD,[3] Renee Jensen RRT,[4] Lena P. Thia MD(Res),[5] Prof Philip Robinson PhD,[6] Prof. Pornchai Tirakitsoontorn, MD,[7] Prof Emeritus Bonnie Ramsey MD,[8] Prof Marcus A. Mall MD,[9, 10, 11] Prof Jennifer L. Taylor-Cousar MD,[1,12] Edward F. McKone MD,[13] Prof Elizabeth Tullis MD,[14] Danieli B. Salinas MD,[15] Jiaqiang Zhu PhD,[15] Yih-Chieh Chen MD,[15] Violeta Rodriguez-Romero PhD,[15] Patrick R. Sosnay MD,[15] Gwyneth Davies MD[16, 17]

[1]University of Colorado School of Medicine and Children's Hospital Colorado, Aurora, CO, US (J E Hoppe, MD); [2]Emory University School of Medicine and Children's Healthcare of Atlanta, Atlanta, GA, US (A S Kasi, MD); [3]Washington University School of Medicine, St. Louis, MO, US (J E Pittman, MD); [4]The Hospital for Sick Children, Toronto, ON, Canada (R Jensen, RRT); [5] Noah's Ark Children's Hospital for Wales, Cardiff, United Kingdom (L P Thia, MD[Res]); [6]The Royal Children's Hospital, Murdoch Children's Institute, and Department of Pediatrics, University of Melbourne, Melbourne, Australia (P Robinson, PhD); [7]Children's Hospital of Orange County and University of California, Irvine, CA, US (P Tirakitsoontorn, MD); [8]Seattle Children's Hospital, University of Washington, Seattle, WA, US (B Ramsey, MD); [9]Department of Pediatric Respiratory Medicine, Immunology and Critical Care Medicine, Charité - Universitätsmedizin Berlin, Berlin, Germany (M A Mall, MD); [10]German Center for Child and Adolescent Health (DZKJ), partner site Berlin, Berlin, Germany (M A Mall, MD); [11]German Center for Lung Research (DZL), associated partner site Berlin, Berlin, Germany (M A Mall, MD); [12]National Jewish Health, Denver, CO, US (J L Taylor-Cousar, MD); [13]St. Vincents University Hospital, University College Dublin, Dublin, Ireland (E F McKone, MD); [14]St Michael's Hospital, University of Toronto, Toronto, ON, Canada (E Tullis, MD); [15]Vertex Pharmaceuticals, Boston, MA, US (D B Salinas, MD; J Zhu, PhD; Y Chen, MD; V Rodriguez-Romero, PhD; P R Sosnay, MD); [16]Population Policy and Practice Department, UCL Great Ormond Street Institute of Child Health, University College London, London, United Kingdom (G Davies, MD); [17]Great Ormond Street Hospital for Children NHS Foundation Trust, London, United Kingdom (G Davies, MD)

Table of Contents

List of Site Investigators 4

Supplementary Methods 5

Trial 121-105 Inclusion Criteria 5

Trial 121-105 Exclusion Criteria 6

121-105 Trial Design 8

Schedule of Assessments 11

Statistical Analysis 13

Adverse Event Definition and Reporting 14

Protocol Deviations 14

Supplementary Figures 15

Figure S1. Steady-state AUC versus Age Group for VNZ, D-IVA, TEZ, and M1-TEZ 15

Supplementary Tables 16

Table S1. Participant Enrollment By Country and Site 16

Table S2. List of IECs/ and IRBs and Approvals 17

Table S3. Additional Baseline Characteristics.* 18

Table S4. Elevated Transaminase Events and Liver Function Test Enzyme Elevations.† 18

Table S5. Summary of Rash Events.* 19

Table S6. Summary of Neuropsychiatric Events.* 19

Table S7. Summary of Blood Pressure Data. 20

Table S8. Absolute Change From Baseline in BMI, Weight, Height, and Associated Z-Scores at Week 24. 21

Table S9. Post hoc analysis of absolute change in $LCI_{2\cdot5}$ by baseline value (≤7·5 units and >7·5 units). 22

Table S10. MMRM Analysis of Absolute Change From Baseline in Sweat Chloride Concentration (mmol/L) at Each Visit up to Week 24 22

Table S11. Post hoc Subgroup Analyses of Absolute Change in Sweat Chloride Concentration From Baseline Through Week 24. 23

Table S12. MMRM Analysis of Absolute Change From Baseline in $ppFEV_1$ (percentage points) at Each Visit up to Week 24 24

Table S13. Post hoc Subgroup Analyses of Absolute Change in $ppFEV_1$ From Baseline Through Week 24. 25

Table S14. Post hoc Subgroup Analyses of Proportion of Participants With Sweat Chloride <60 mmol/L Through Week 24 26

Table S15. Post hoc Subgroup Analyses of Proportion of Participants With Sweat Chloride <30 mmol/L Through Week 24 26

References 27

List of Site Investigators

The VX21-121-105 Study Group included Gary McPhail, Cincinnati Children’s Hospital Medical Center, Cincinnati, Ohio; Karen McCoy, Nationwide Children’s Hospital, Columbus, Ohio; Erica Roesch, University Hospitals Rainbow Babies and Children's Hospital, Cleveland, Ohio; Daniel Weiner, UPMC Children's Hospital of Pittsburgh, Pittsburgh, Pennsylvania; Carlos Milla, Leland Stanford Junior University, Palo Alto, California; Jessica Pittman, Washington University, St. Louis, Missouri; Rachel Linnemann, Emory University, Atlanta, Georgia; Anne Stone, Oregon Health & Science University, Portland, Oregon; Hugo Escobar, The Children’s Mercy Hospital, Kansas City, Missouri; James Chmiel, Riley Hospital for Children at Indiana University, Indianapolis, Indiana; Fadel Ruiz, Texas Children’s Hospital – Baylor College of Medicine, Houston, Texas; Jonathan Gaffin, Boston Children’s Hospital, Boston, Massachusetts; Hara Levy, American Family Children's Hospital, Madison, Wisconsin; Thomas Lahiri, University of Vermont Medical Center, Colchester, Vermont; Jordana Hoppe, Children’s Hospital Colorado, Aurora, Colorado; Brooke Moore, Children's Respiratory and Critical Care Specialists, Minneapolis, Minnesota; Gwyneth Davies, Great Ormond Street Hospital, London, United Kingdom; Alexander Moeller, Kinderspital Zuerich, Zurich, Switzerland; Anna-Maria Dittrich, Medizinische Hochschule Hannover, Hannover, Germany; Philipp Latzin, Inselspital - Universitaetsspital Bern, Bern, Switzerland; Pornchai Tirakitsoontorn, Children's Hospital of Orange County, Orange, California; Maria Dowell, Ann & Robert H. Lurie Children's Hospital of Chicago, Chicago, Illinois; Lena Thia, Cardiff and Vale University Health Board, Cardiff, United Kingdom; Mirjam Stahl, Charite Universitätsmedizin Berlin, Berlin, Germany; Florian Stehling, Universitaetsklinikum Essen Kinderklinik III, Abt. fuer Pneumologie, Essen, Germany; Joan DeCelie-Germana, Northwell Health-Cohen Children's Medical Center, Lake Success, New York; Isabelle Sermet-Gaudelus, Hopital Necker, Enfants Malade, Paris, France; Hettie Janssens, Erasmus Medical Center / Sophia Children's Hospital, Rotterdam, Netherlands; Philippe Reix, CHU Lyon - Hopital Femme Mere-Enfant, Bron Cedex, France; Philip Robinson, The Royal Children’s Hospital, Victoria, Australia; Claire Wainwright, Queensland Children’s Hospital, Queensland,Australia; Andrew Tai, Women’s and Children’s Hospital, North Adelaide, Australia; Marcus Svedberg, Sahlgrenska Universitetssjukhuset, Göteborg, Sweden

Supplementary Methods

The trial was conducted in accordance with the Declaration of Helsinki, local applicable laws and regulations, and current Good Clinical Practice (GCP) Guidelines as described by the International Council for Harmonization.

Trial 121-105 Inclusion Criteria

1. Participant (or participant's legally appointed and authorized representative) signed and dated an informed consent form (ICF), and an assent form.
2. Participants 6 through 11 years of age (inclusive), on the date of informed consent; participants who completed Cohort A1 but are ≥12 years of age on the date of informed consent in Cohort B1 are not eligible to enrol.
3. Participants whose weight (without shoes) was between the 5th and 95th percentile (inclusive) for weight-for-age at the Screening Visit based on current Center for Disease Control (CDC) growth charts.
4. Confirmed diagnosis of cystic fibrosis (CF) as determined by the investigator.
5. Participants had at least 1 triple combination-responsive (TCR) mutation (including *F508del*) in the cystic fibrosis transmembrane receptor (*CFTR*) gene. Genotype should be confirmed at the screening visit. If the screening *CFTR* genotype result is not received before the first dose of study drug, a previous *CFTR* genotype laboratory report may be used to establish eligibility.
    - Participant who has been enrolled and whose screening genotype does not confirm trial eligibility must be discontinued from the trial.
6. Participant with forced expiratory volume in 1 second (FEV_1) ≥60% of predicted normal for age, sex, and height using equations of the Global Lung Function Initiative (GLI)[1] at the Screening Visit. Spirometry measurements used to confirm eligibility must meet American Thoracic Society/European Respiratory Society criteria[2] for acceptability and repeatability, as judged by the investigator.
7. Participant with stable CF disease at the start of the treatment period as deemed by the investigator.
8. Participant willing to remain on a stable CF medication regimen (other than CFTR modulators) through week 24 or, if applicable, through the Safety Follow-up Visit.
9. Participants who was able to swallow tablets.

10. Female participant of childbearing potential must have had a negative serum pregnancy test at the Screening Visit.

11. Participant of childbearing potential and who was sexually active must have met the contraception requirements.

12. As deemed by the investigator, the participant's legally appointed and authorized representative (e.g., parent or legal guardian) AND the participant must have been able to understand protocol requirements, restrictions, and instructions. The participant's legally appointed and authorized representative ensured that the participant would comply with and was likely to complete the trial as planned.

Trial 121-105 Exclusion Criteria

1. History of any illness or any clinical condition that, in the opinion of the investigator, might have confounded the results of the trial or posed an additional risk in administering study drug to the participant. This included, but was not limited to, the following:
    - Hepatic cirrhosis with portal hypertension, moderate hepatic impairment (Child Pugh Score 7 to 9), or severe hepatic impairment (Child Pugh Score 10 to 15)
    - Chronic kidney disease of Stage 3 or above
    - Solid organ or hematological transplantation
    - Alcohol or drug abuse in the past year, including, but not limited to, cannabis, cocaine, and opiates, as deemed by the investigator
    - Cancer
2. Any clinically significant laboratory abnormalities at the Screening Visit that would have interfered with the trial assessments or posed an undue risk for the participant (as deemed by the investigator).

3. History of intolerance to study drug that would have posed an additional risk to the participant in the opinion of the investigator (e.g., participants with a history of liver function test [LFT] elevations requiring treatment interruption or discontinuation, allergy or hypersensitivity to the study drug).

4. Any of the following abnormal laboratory values at screening:
    - Haemoglobin <10 g/dL
    - Total bilirubin ≥2 × upper limit of normal (ULN)
    - Aspartate transaminase (AST), alanine transaminase (ALT), γ-glutamyl transferase (GGT), or alkaline phosphatase (ALP) ≥3 × ULN
    - Abnormal renal function defined as glomerular filtration rate ≤45 mL/min/1·73 m^2 (calculated by the Counahan-Barratt equation)[3]
5. An acute upper or lower respiratory infection, pulmonary exacerbation (PEx), or changes in therapy (including antibiotics) for pulmonary disease within 28 days before day 1 (first dose of study drug).
6. Lung infection with organisms associated with a more rapid decline in pulmonary status (including, but not limited to, *Burkholderia cenocepacia*, *Burkholderia dolosa*, and *Mycobacterium abscessus*). For participants who had a history of a positive culture, the investigator applied the following criteria to establish whether the participant was free of infection with such organisms:
    - The participant had not had a respiratory tract culture positive for these organisms within the 12 months before the date of informed consent and assent.
    - The participant had at least 2 respiratory tract cultures negative for such organisms within the 12 months before the date of informed consent, with the first and last of these separated by at least 3 months, and the most recent one within the 6 months before the date of informed consent and assent.
7. An acute illness not related to CF (e.g., gastroenteritis) within 14 days before day 1 (the first dose of vanzacaftor/tezacaftor/deutivacaftor [VNZ/TEZ/D-IVA]).
8. Ongoing or prior participation in a trial of an investigational treatment other than a Vertex CFTR modulator within 28 days or 5 terminal half-lives (whichever is longer) before screening, or participation in an interventional trial of a non-investigational treatment from screening through end of trial participation. The duration of the elapsed time may have been longer if required by local regulations. Note: Ongoing participation in a noninterventional trial (including observational trials) was permitted.
9. Use of restricted medication within specified duration before the first dose of study drug.

10. The participant or a close relative of the participant was the investigator or a subinvestigator, research assistant, pharmacist, trial coordinator, or other staff directly involved with the conduct of the trial.

121-105 Trial Design

Run-In Period

The run-in period had a 4-week duration and was designed to establish a reliable on-treatment (elexacaftor/tezacaftor/ivacaftor [ELX/TEZ/IVA]) baseline for the treatment period. The first dose of open-label ELX/TEZ/IVA was administered at the day -28 visit. The last dose of open-label ELX/TEZ/IVA was administered on day -1 (1 day before the day 1 visit).

- Participants received either of the following ELX/TEZ/IVA doses based on their weight at day -28: Participants weighing <30 kg: ELX 100 mg once daily (qd)/TEZ 50 mg qd/IVA 75 mg every 12 hours (q12h)
- Participants weighing ≥30 kg: ELX 200 mg qd/TEZ 100 mg qd/IVA 150 mg q12h

Participants who were currently receiving stable ELX/TEZ/IVA treatment had the run-in period waived and entered the treatment period within 28 days following the Screening Visit. Participants who prematurely discontinued ELX/TEZ/IVA during the run-in period did not participate in the treatment period.

Treatment Period

Participants received open-label VNZ/TEZ/D-IVA for 24 weeks based on their weight at day 1:

- Participants weighing <40 kg: VNZ 12 mg qd/TEZ 60 mg qd/D-IVA 150 mg qd, administered as 3 fixed-dose combination tablets
- Participants weighing ≥40 kg: VNZ 20 mg qd/TEZ 100 mg qd/D-IVA 250 mg qd, administered as 2 fixed-dose combination tablets

Follow-up

A safety follow-up visit was scheduled to occur 28 (± 7) days after the last dose of study drug for participants who completed study drug dosing and for participants who prematurely discontinued study drug dosing. The safety follow-up visit was not required for participants who completed the treatment period and transitioned within 28 days of the last dose of study drug to either:

- a commercially available Vertex CFTR modulator regimen,
- a managed access program-supplied Vertex CFTR modulator regimen,
- or, an open-label study or other qualified Vertex study.

If a participant prematurely discontinued treatment, an Early Termination of Treatment (ETT) Visit was to be scheduled as soon as possible after the decision to discontinue treatment. Participants who prematurely discontinued treatment were also required to complete the Safety Follow-up Visit, as above. Participants who prematurely discontinued treatment were not eligible to enroll in the open-label extension safety study.

If a participant withdrew consent for the trial, no further assessments were performed.

Treatment Interruption and Stopping Rules

Modifications of the study drug dose were prohibited. Treatment may have been interrupted as outlined below. If any unacceptable toxicity arose, individual participants were to discontinue dosing.

Liver function tests

The central laboratory notified the medical monitor of ALT or AST >3 × ULN and total bilirubin >2 × ULN that were derived from centrally submitted samples.

Participants with new treatment-emergent ALT or AST elevations of >3 × ULN, with or without total bilirubin >2 × ULN, were followed closely, including confirmatory testing performed by the central laboratory within 48 to 72 hours of the initial finding and subsequent close monitoring of ALT, AST, and bilirubin levels, as clinically indicated.

Study drug administration was interrupted immediately (prior to confirmatory testing) if any of the following criteria were met:

- ALT or AST >8 × ULN
- ALT or AST >5 × ULN for more than 2 weeks
- ALT or AST >3 × ULN, in association with total bilirubin >2 × ULN and/or clinical jaundice

A thorough investigation of potential causes was to be conducted, and the participant was followed closely for clinical progression.

Study drug administration was discontinued if the following criterion was met:

- Subsequent ALT or AST values confirmed the initial elevation that satisfied the interruption rule (above), and no convincing alternative etiology (e.g., acetaminophen use, viral hepatitis, alcohol ingestion) was identified, regardless of whether transaminase levels had improved

All participants in whom treatment was discontinued for elevated transaminases (and bilirubin, as applicable) was to have these levels monitored closely until levels normalized or returned to baseline.

If an alternative, reversible cause of transaminase elevation with or without increased bilirubin or clinical jaundice was identified, study drug administration may have been resumed once transaminases returned to baseline or were ≤2 × ULN, whichever was higher. Upon resumption of study drug, transaminases and bilirubin were to be assessed weekly for 4 weeks. If a protocol-defined transaminase elevation interruption threshold recurred within 4 weeks of rechallenge with the study drug (with confirmation of the initial elevation by repeat testing within 48 to 72 hours), then the study drug was permanently discontinued, regardless of the presumed etiology.

Rash

Participants who developed a generalized rash were monitored closely. Study drug dosing was to be interrupted if a participant developed a generalized rash of Grade 3 or higher, or a rash that was considered a serious adverse event. The participant may have resumed study drug treatment if considered clinically appropriate by the investigator.

Schedule of Assessments

Trial VX21-121-105: Part B Run-in Period, Treatment Period, and Safety Follow-up Visit (Participants 6 Through 11 Years of Age)

	**Run-in Period**		**Treatment Period**							**Safety Follow-up Visit**
**Event/Assessment[1]**	**Day -28 (± 1 Day)**	**Day -14 (Day -15 to Day -3)**	**Day 1**	**Day 3 (± 1 Day)**	**Day 15 (± 3 Days)**	**Weeks 4, 8, 12, and 16 (± 5 Days)**	**Week 20 (± 5 Days)**	**Week 24 (± 5 Days)**	**ETT Visit**	**28 (± 7) Days After the Last Dose of Study Drug**
CFQ-R[2]			X			X		X	X	X
Full physical examination	X		X			Week 12		X	X	
Weight and height	X		X		X	X		X	X	X
Vital signs	X		X		X	X		X	X	X
Pulse oximetry	X		X		X	X		X	X	X
Standard 12-lead ECG	X		X (triplicate)		X	Week 12		X	X	X
Pregnancy test	Serum		Serum			Serum	Urine	Serum	Serum	Serum
Serum chemistry[3]	X		X		X	X		X	X	X
Hematology[4]	X		X		X	X		X	X	X
Coagulation[4]	X		X					X	X	X
Urinalysis[4]	X		X					X	X	
Multiple-breath washout[4]			X		X	Weeks 4 and 12		X	X	
Spirometry[5]			X		X	X		X	X	X
Sweat chloride[6]			X		X	Weeks 4 and 16		X	X	
Fecal sample collection			X		X	Weeks 4 and 12		X	X	
IRT			X		X	Weeks 4 and 12		X	X	
PK sampling[7]			X		X	X				

[1] All assessments were performed before study drug dosing unless noted otherwise.
[2] MCID is based on external literature.[5]
[3] Samples were analyzed at a central laboratory.
[4] LCI value was calculated by a central reader.
[5] All sites were provided with spirometers for study assessments and data were transmitted to a centralized spirometry service for quality review.
[6] Sweat chloride was collected with an approved collection device and sent to a central laboratory for testing and interpretation.
[7] **Day 1**: predose and 2 h postdose

Day 15 and Week 4: predose

Week 8: predose, and 2.5 and 3.5 h postdose for all subjects. For a minimum of 12 subjects who weigh ≥17 kg at Screening, additional 6.5 and 8 h postdose samples were collected.

Weeks 12 and 16: predose

Trial VX21-121-105: Part B Run-in Period, Treatment Period, and Safety Follow-up Visit (Participants 6 Through 11 Years of Age)

	Run-in Period		Treatment Period							
**Event/Assessment[1]**	**Day -28 (± 1 Day)**	**Day -14 (Day -15 to Day -3)**	**Day 1**	**Day 3 (± 1 Day)**	**Day 15 (± 3 Days)**	**Weeks 4, 8, 12, and 16 (± 5 Days)**	**Week 20 (± 5 Days)**	**Week 24 (± 5 Days)**	**ETT Visit**	**Safety Follow-up Visit 28 (± 7) Days After the Last Dose of Study Drug**
ELX/TEZ/IVA dosing	Day -28 through Day -1									
VNZ/TEZ/D-IVA dosing			Day 1 through day before Week 24 Visit							
Other events related to outcome	Continuous from signing of ICF (and assent form) through completion of trial participation									
Medications review	Continuous from signing of ICF (and assent form) through completion of trial participation									
Non-pharmacological treatments and procedures review	Continuous from signing of ICF (and assent form) through completion of trial participation									
AEs and SAEs	Continuous from signing of ICF (and assent form) through completion of trial participation									

AE: adverse event; CFQ-R: Cystic Fibrosis Questionnaire – Revised; D-IVA: deutivacaftor; ECG: electrocardiogram; ELX: elexacaftor; ETT: early termination of treatment; ICF: informed consent form; IRT: immunoreactive trypsinogen; IVA: ivacaftor; MCID: minimum clinically important difference; PK: pharmacokinetic; SAE: serious adverse event; TEZ: tezacaftor; VNZ: vanzacaftor

Statistical Analysis

Safety analysis

Safety data was analysed descriptively; no statistical hypothesis testing was performed. Adverse events, including neuropsychiatric events, were collected during the trials and reported by the investigator as per the protocol. There were no formal mental health assessments conducted in Trial 121-105.

Efficacy

PEx were defined as a new or change in antibiotic therapy (intravenous [IV], inhaled, or oral) for any 4 or more of the following signs/symptoms. This definition was based on the definition of a PEx used in previous clinical studies, including IVA clinical studies.[6,7]

- Change in sputum
- New or increased hemoptysis
- Increased cough
- Increased dyspnea
- Malaise, fatigue, or lethargy
- Temperature above 38°C (equivalent to approximately 100.4°F)
- Anorexia or weight loss
- Sinus pain or tenderness
- Change in sinus discharge
- Change in physical examination (PE) of the chest
- Decrease in pulmonary function by 10%
- Radiographic changes indicative of pulmonary infection

Multiplicity: Not applicable since no formal hypotheses was tested.

Population PK

Population PK analyses including data from the pivotal Phase 3 trials 121-102 and 121-103 (subjects ≥12 years of age) and from Trial 121-105 (subjects 6 through 11 years of age) identified weight as the key covariate having a clinically meaningful impact on VNZ, TEZ, M1-TEZ, and D-IVA disposition and informed dose selection for the 6- through 11-year-old paediatric population. VNZ, TEZ, M1-TEZ and D-IVA population PK models described the PK data reasonably well.

Adverse Event Definition and Reporting

All participants or participants' parents or legal guardians were queried, using nonleading questions, about the occurrence of AEs at each trial visit. When possible, a constellation of signs and/or symptoms were identified as 1 overall event or diagnosis.

An AE was defined as any untoward medical occurrence in a participant during the trial; the event did not necessarily have a causal relationship with the treatment. This included any newly occurring event or worsening of a pre-existing condition (e.g., increase in its severity or frequency) after the ICF is signed.

The investigator determined and recorded the severity (mild, moderate, severe, life-threatening, death) of all serious and nonserious AEs. The guidance available at the following website was consulted: Common Terminology Criteria for Adverse Events (CTCAE), Version 5.0, Cancer Therapy Evaluation Program, http://ctep.cancer.gov/protocolDevelopment/electronic_applications/ctc.htm (Accessed August 2022). When considering the severity of an AE in a pediatric subject, the investigator was to consider that reference ranges for pediatric clinical laboratory parameters may differ from those in the CTCAE.

Protocol Deviations

An important protocol deviation (IPD) was defined as any protocol deviation that may have significantly impacted the completeness, accuracy, or reliability of key trial data or that may have significantly affected a subject's rights, safety, or well-being.

A total of 2 participants had an IPD related to investigational product.

Supplementary Figures

Figure S1. Steady-state AUC versus Age Group for VNZ, D-IVA, TEZ, and M1-TEZ

Panel A) VNZ

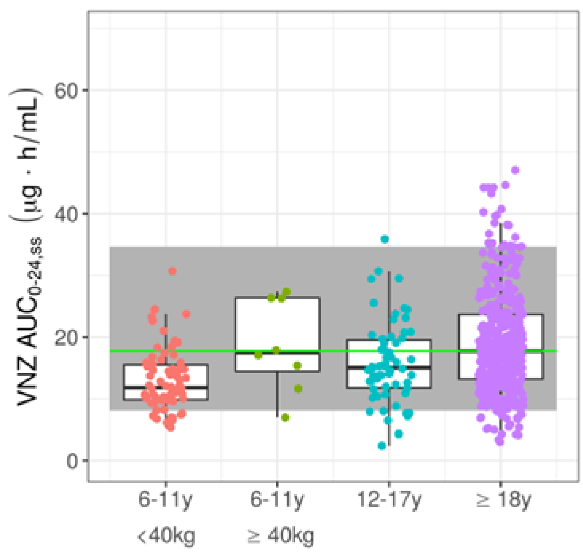

Panel B) D-IVA

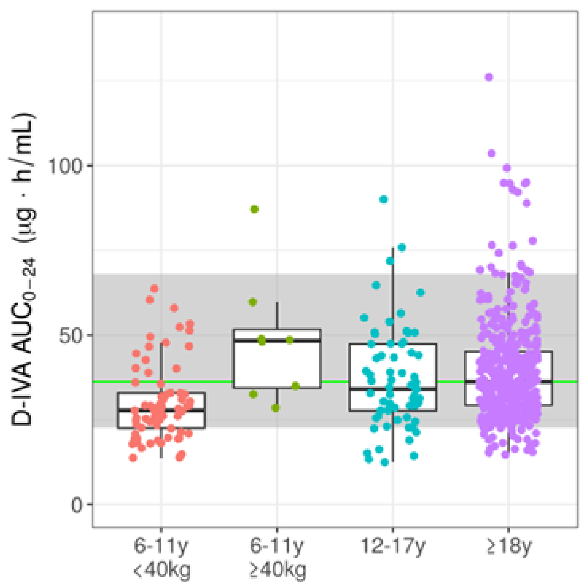

Panel C) TEZ

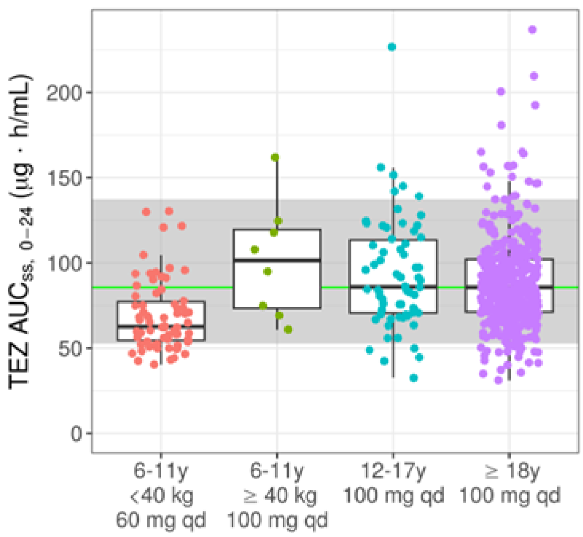

Panel D) M1-TEZ

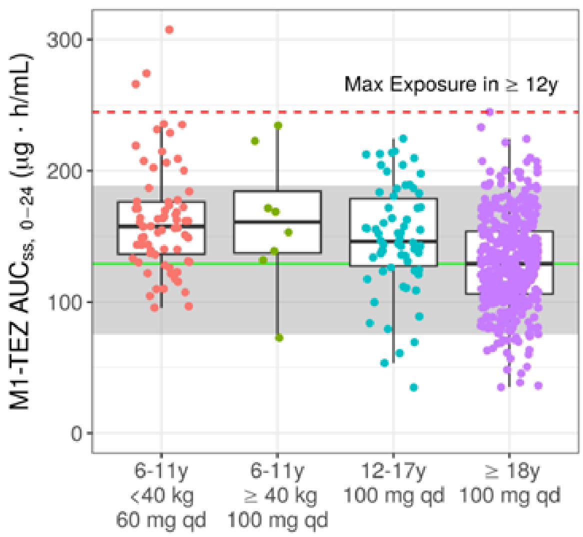

$AUC_{0\text{-}24h}$: AUC from the time of dosing to 24 hours; D-IVA: deutivacaftor; EBE: empirical Bayes estimate; IQR: interquartile range; qd: once daily; TEZ: tezacaftor; VNZ: vanzacaftor; y: years of age

Notes: Adults and adolescents received VNZ 20 mg/TEZ 100 mg/D-IVA 250 mg qd dose, children 6 through 11 years of age ≥40 kg received VNZ 20 mg/TEZ 100 mg/D-IVA 250 mg qd dose, and children 6 through 11 years of age <40 kg received VNZ 12 mg/TEZ 60 mg/D-IVA 150 mg qd dose. Green horizontal line represents the median of the adult values and the grey shaded area indicates the 5th and 95th percentiles of the adult values. Points represent individual EBE values. Boxplots present statistics of the points, with the median represented by a horizontal line, and the IQR represented by a box. The whiskers mark the minimum and maximum values within 1.5 × IQR from Q1 and Q3, respectively.

Supplementary Tables

Table S1. Participant Enrollment By Country and Site

Country	Site	Number of Participants Enrolled
United States	Cincinnati Children’s Hospital Medical Center	2
	Nationwide Children’s Hospital	1
	University Hospitals Rainbow Babies and Children’s Hospital	2
	UPMC Children’s Hospital of Pittsburgh	3
	Leland Stanford Junior University	3
	Washington University	4
	Emory University	2
	The Children’s Mercy Hospital	4
	Riley Hospital for Children at Indiana University	2
	Texas Children’s Hospital – Baylor College of Medicine	2
	Boston Children’s Hospital	3
	American Family Children’s Hospital	1
	University of Vermont Medical Center	2
	Children’s Hospital Colorado	6
	Children’s Respiratory and Critical Care Specialists	2
	Children’s Hospital of Orange County	2
	Ann & Robert H. Lurie Children’s Hospital of Chicago	4
	Northwell Health-Cohen Children’s Medical Center	2
United Kingdom	Great Ormond Street Hospital for Children	3
	Cardiff and Vale University Health Board	2
Switzerland	Kinderspital Zurich	2
	Inselspital – Universitaetsspital Bern	1
Germany	Medizinische Hochschule Hannover	3
	Charite Universitätsmedizin Berlin	2
	Universitaetsklinikum Essen Kinderklinik III, Abt. Fuer Pneumologie	3
France	Hopital Necker, Enfants Malades	2
	CHU Lyon – Hopital Femme Mere-Enfant	2
Netherlands	Erasmus Medical Center / Sophia Children’s Hospital	2
Australia	The Royal Children’s Hospital	4
	Queensland Children’s Hospital	3
	Women’s and Children’s Hospital	1
Sweden	Sahlgrenska Universitetssjukhuset	1

Table S2. List of IECs/ and IRBs and Approvals

Country	Institutional Review Board/Independent Ethics Committee	Approval Number
United States	Advarra, Inc.	MOD01379147
	Committee on Clinical Investigation	Protocol number IRB-P0041583
	University of Vermont-Research Protection Office	STUDY00002300
	Children’s Minnesota IRBs	iRIS Reference #: 006883
United Kingdom	West Midlands-Coventry & Warwickshire Research Ethics Committee	22/WM/0239
Swtizerland	Kantonale Ethikkommission Zurich	BASEC no. 2022-01673
Germany	Ethikkommission der Universitaet Duisburg-Essen	22-10932-AF
France	CPP Sud Ouest et Outre Mer I ARS Midi Pyrénées	1-22-080 / 22.03687.000164
Netherlands	Medisch Ethische Toetsings Commissie Erasmus MC	MEC-2022-0630
Australia	Children’s Health Queensland Hospital and Health Service Human Research Ethics Committee	HREC/22/QCHQ/89003
Sweden	Etikprövningsmyndigheten	2022-04960-01

Table S3. Additional Baseline Characteristics.*

	VNZ/TEZ/D-IVA (N=78) n (%)
***Pseudomonas aeruginosa* infection within 2 years prior to screening**	
Positive	10 (12·8)
Negative	68 (87·2)
**FE-1 (mg/kg)**	
Mean (SD)	133·9 (188·6)
**$LCI_{2.5}$**	
Mean (SD)	6·63 (0·74)

CFTR: cystic fibrosis transmembrane conductance regulator; n: size of subsample; N: total sample size; VNZ/TEZ/D-IVA: vanzacaftor/tezacaftor/deutivacaftor

* Baseline characteristics of the full analysis set, which was defined as all randomised children who carried the intended *CFTR* mutations and received ≥1 dose of study drug in the treatment period. Baseline was defined as the most recent non-missing measurement before the first dose of study drug in the treatment period.

† Prior use is defined as anytime within 56 days before the date of first dose in the treatment period, defined as the first dose of ELX/TEZ/IVA, IVA, or TEZ/IVA after randomization. This does not include IVA or TEZ administered during the run-in period.

Table S4. Elevated Transaminase Events and Liver Function Test Enzyme Elevations.†

	VNZ/TEZ/D-IVA N=78 n (%)
**Any elevated transaminase events**	**4 (5·1)**
Alanine aminotransferase increased	4 (5·1)
Aspartate aminotransferase increased	2 (2·6)
**Maximum severity of adverse event**	
Mild	3 (3·8)
Moderate	1 (1·3)
**Adverse events leading to discontinuation of study drug**	**0**
**Adverse events leading to interruption of study drug**	**0**
**Serious adverse events**	**0**
**ALT or AST***	
>3 × ULN	3 (3·8)
>5 × ULN	1 (1·3)
>8 × ULN	0
ALT or AST >3 × ULN and total bilirubin >2 × ULN*	0

ALT: alanine transaminase; AST: aspartate transaminase; n: size of subsample; N: total sample size; ULN: upper limit of normal; VNZ/TEZ/D-IVA: vanzacaftor/tezacaftor/deutivacaftor

* For the liver function test threshold analyses, each percentage is calculated as (n/N1)×100, where the numerator n is the number of children meeting the indicated threshold and the denominator (N1) is the number of children with at least 1 non-missing measurement during the treatment-emergent period. For "ALT or AST", counts are based on the highest value of either test during the treatment-emergent period for each child. A child whose highest value is >5×ULN is also counted as >3×ULN. A child whose highest value is >8×ULN is also counted as >3×ULN and >5×ULN.

† Group term of "elevated transaminase events" included multiple preferred terms; those that actually occurred in this trial were alanine aminotransferase increased and aspartate aminotransferase increased.

Table S5. Summary of Rash Events.*

	VNZ/TEZ/D-IVA<br>N=78
**Children with any rash events, n (%)**	**4 (5·1)**
Rash	4 (5·1)
**Children with rash events by maximum severity, n (%)**	
Mild	4 (5·1)
**Children with adverse events leading to discontinuation of study drug, n (%)**	**0**
**Children with adverse events leading to interruption of study drug, n (%)**	**0**
**Children with serious adverse events, n (%)**	**0**
**Time-to-onset of first event (days)**	
Mean (SD)	8·0 (3·8)

n: size of subsample; N: total sample size; VNZ/TEZ/D-IVA: SD: standard deviation; vanzacaftor/tezacaftor/deutivacaftor

* When summarizing numbers and percentages of children, a child with multiple events within a category is counted only once in that category. Group term of "rash events" includes terms of rash (e.g., rash, rash erythematous, rash maculopapular, rash papular, skin exfoliation, and urticaria).

Table S6. Summary of Neuropsychiatric Events.*

	VNZ/TEZ/D-IVA<br>N=78<br>n (%)
**Any neuropsychiatric events**	**4 (5·1)**
Initial insomnia	2 (2·6)
Aggression	1 (1·3)
Anxiety	1 (1·3)
**Maximum severity of adverse event**	
Mild	4 (5·1)
**Adverse events leading to discontinuation of study drug**	**0**
**Adverse events leading to interruption of study drug**	**0**
**Serious adverse events**	**0**

n: size of subsample; N: total sample size; VNZ/TEZ/D-IVA: vanzacaftor/tezacaftor/deutivacaftor

* When summarizing numbers and percentages of participants, a participant with multiple events within a category is counted only once in that category. Group term of "neuropsychiatric events" included multiple Preferred Terms, including depression/suicidality, anxiety, insomnia, behavioural, mental fatigue, etc.

Table S7. Summary of Blood Pressure Data.

	VNZ/TEZ/D-IVA N=78	
**Mean (SD), mmHg**	**SBP**	**DBP**
Baseline*	105·1 (10·9)	63·8 (8·2)
Δ day 15	-0·6 (9·2)	-0·8 (9·2)
Δ week 4	-1·4 (10·8)	-0·6 (8·6)
Δ week 8	-1·6 (9·0)	-0·7 (9·5)
Δ week 12	-1·2 (9·5)	-1·5 (8·2)
Δ week 16	-2·6 (10·9)	-1·7 (10·4)
Δ week 24	-1·8 (11·5)	1·1 (9·9)

DBP: diastolic blood pressure; N: total sample size; SBP: systolic blood pressure; SD: standard deviation; VNZ/TEZ/D-IVA: vanzacaftor/tezacaftor/deutivacaftor

* Baseline was defined as the most recent non-missing measurement before the first dose of study drug in the treatment period.

Table S8. Absolute Change From Baseline in BMI, Weight, Height, and Associated Z-Scores at Week 24.

Parameter	VNZ/TEZ/D-IVA N=78
**BMI (kg/m^2)**	
**Baseline**	
n	78
Mean (SD)	16·83 (2·13)
**Absolute change at week 24**	
n	78
LS mean	0·22
95% CI of LS mean	(0·05, 0·38)
**BMI z-score**	
**Baseline**	
n	78
Mean (SD)	0·07 (0·87)
**Absolute change at week 24**	
n	78
LS mean	-0·05
95% CI of LS mean	(-0·12, 0·02)
**Weight (kg)**	
**Baseline**	
n	78
Mean (SD)	30·21 (7·48)
**Absolute change at week 24**	
n	78
LS mean	1·67
95% CI of LS mean	(1·34, 2·00)
**Weight z-score**	
**Baseline**	
n	78
Mean (SD)	0·00 (0·89)
**Absolute change at week 24**	
n	78
LS mean	-0·02
95% CI of LS mean	(-0·07, 0·03)
**Height (cm)**	
**Baseline**	
n	78
Mean (SD)	133·0 (10·5)
**Absolute change at week 24**	
n	78
LS mean	2·7
95% CI of LS mean	(2·5, 3·0)
**Height z-score**	
**Baseline**	
n	78
Mean (SD)	-0·03 (0·94)
**Absolute change at week 24**	
n	78
LS mean	0·01
95% CI of LS mean	(-0·03, 0·05)

BMI: body mass index; CI: confidence interval; LS: least squares; n: size of subsample; N: total sample size; SD: standard deviation; VNZ/TEZ/D-IVA: vanzacaftor/tezacaftor/deutivacaftor

Note: Baseline was defined as the most recent non-missing measurement (scheduled or unscheduled) collected before the first dose of VNZ/TEZ/D-IVA in the treatment period.

Table S9. Post hoc analysis of absolute change in $LCI_{2\cdot5}$ by baseline value (≤7·5 units and >7·5 units).

	Parameter	Baseline $LCI_{2\cdot5}$ ≤7·5	Baseline $LCI_{2\cdot5}$ >7·5
**Baseline**	**n**	**62**	**10**
	Mean (SD)	6·40 (0·46)	8·04 (0·60)
**Absolute change at week 12**	**n**	**54**	**9**
	Mean (SD)	0·03 (0·52)	-0·12 (0·64)
**Absolute change at week 24**	**n**	**50**	**8**
	Mean (SD)	-0·06 (0·48)	-0·82 (0·47)

$LCI_{2\cdot5}$: number of lung turnovers required to reduce the end tidal inert gas concentration to 1/40th of its starting value; n: size of subsample; SD: standard deviation

Note: Baseline was defined as the pre-dose day 1 value. For participants who were on stable ELX/TEZ/IVA (i.e., did not participate in the run-in period), if the pre-dose day 1 value was missing, screening assessments were used as baseline.

Table S10. MMRM Analysis of Absolute Change From Baseline in Sweat Chloride Concentration (mmol/L) at Each Visit up to Week 24

	**VNZ/TEZ/D-IVA<br>N = 78**
**Baseline**	
n	77
Mean (SD)	40.4 (20.9)
**Absolute change at day 15**	
n	72
LS mean (SE)	-5.9 (1.3)
95% CI of LS mean	(-8.6, -3.3)
**Absolute change at week 4**	
n	70
LS mean (SE)	-6.8 (1.3)
95% CI of LS mean	(-9.4, -4.2)
**Absolute change at week 16**	
n	76
LS mean (SE)	-8.8 (1.6)
95% CI of LS mean	(-11.9, -5.6)
**Absolute change at week 24**	
n	74
LS mean (SE)	-8.4 (1.2)
95% CI of LS mean	(-10.8, -6.1)

D-IVA: deutivacaftor; LS: least squares; MMRM: mixed-effects model for repeated measures; n: size of subsample; N: total sample size; TEZ: tezacaftor; VNZ: vanzacaftor

Note: Baseline was defined as the pre-dose day 1 value. For participants who were on stable ELX/TEZ/IVA (i.e., did not participate in the run-in period), if the pre-dose day 1 value was missing, the most recent available predose value, including the screening assessment, was used as baseline.

Table S11. Post hoc Subgroup Analyses of Absolute Change in Sweat Chloride Concentration From Baseline Through Week 24.

	**VNZ/TEZ/D-IVA N=78**
**Genotype Subgroup: *F508del-F508del***	
**Baseline**	
n	37
Mean (SD)	38·4 (21·3)
**Absolute change through week 24**	
n	37
LS mean	-9·3
95% CI of LS mean	(-13·2, -5·5)
**Genotype Subgroup: *F508del*-minimal function**	
**Baseline**	
n	23
Mean (SD)	52·6 (18·9)
**Absolute change through week 24**	
n	23
LS mean	-10·5
95% CI of LS mean	(-14·8, -6·3)
**Genotype Subgroup: Other Genotypes**	
**Baseline**	
n	17
Mean (SD)	28·4 (13·9)
**Absolute change through week 24**	
n	17
LS mean	-4·7
95% CI of LS mean	(-9·3, 0·0)

CI: confidence interval; LS: least squares; n: size of subsample; N: total sample size; SD: standard deviation; VNZ/TEZ/D-IVA: vanzacaftor/tezacaftor/deutivacaftor

Note: Baseline was defined as the pre-dose day 1 value. For participants who were on stable ELX/TEZ/IVA (i.e., did not participate in the run-in period), if the pre-dose day 1 value was missing, screening assessments were used as baseline.

Table S12. MMRM Analysis of Absolute Change From Baseline in $ppFEV_1$ (percentage points) at Each Visit up to Week 24

	VNZ/TEZ/D-IVA<br>N = 78
**Baseline**	
n	77
Mean (SD)	99.7 (15.1)
**Absolute change at day 15**	
n	71
LS mean (SE)	-1.6 (0.9)
95% CI of LS mean	(-3.5, 0.2)
**Absolute change at week 4**	
n	64
LS mean (SE)	-1.1 (0.8)
95% CI of LS mean	(-2.7, 0.6)
**Absolute change at week 8**	
n	72
LS mean (SE)	-1.5 (1.3)
95% CI of LS mean	(-4.1, 1.1)
**Absolute change at week 12**	
n	72
LS mean (SE)	0.1 (1.0)
95% CI of LS mean	(-1.9, 2.1)
**Absolute change at week 16**	
n	73
LS mean (SE)	0.2 (1.1)
95% CI of LS mean	(-2.0, 2.5)
**Absolute change at week 24**	
n	69
LS mean (SE)	-0.3 (1.1)
95% CI of LS mean	(-2.4, 1.8)

D-IVA: deutivacaftor; LS: least squares; MMRM: mixed-effects model for repeated measures; n: size of subsample; N: total sample size; $ppFEV_1$: percent predicted forced expiratory volume in 1 second; TEZ: tezacaftor; VNZ: vanzacaftor

Note: Baseline was defined as the pre-dose day 1 value. For participants who were on stable ELX/TEZ/IVA (i.e., did not participate in the run-in period), if the predose day 1 value was missing, the most recent available predose value, including the screening assessment, was used as baseline.

Table S13. Post hoc Subgroup Analyses of Absolute Change in ppFEV$_1$ From Baseline Through Week 24.

	VNZ/TEZ/D-IVA N=78
**Genotype Subgroup: *F508del-F508del***	
**Baseline**	
n	37
Mean (SD)	101·9 (11·3)
**Absolute change through week 24**	
n	36
LS mean	-0·3
95% CI of LS mean	(-3·4, 2·8)
**Genotype Subgroup: *F508del*-minimal function**	
**Baseline**	
n	24
Mean (SD)	94·3 (21·3)
**Absolute change through week 24**	
n	22
LS mean	2·6
95% CI of LS mean	(-1·9, 7·1)
**Genotype Subgroup: Other Genotypes**	
**Baseline**	
n	16
Mean (SD)	102·5 (9·1)
**Absolute change through week 24**	
n	16
LS mean	-1·9
95% CI of LS mean	(-6·2, 2·5)

CI: confidence interval; LS: least squares; n: size of subsample; N: total sample size; ppFEV$_1$: percent predicted forced expiratory volume in 1 second; SD: standard deviation; VNZ/TEZ/D-IVA: vanzacaftor/tezacaftor/deutivacaftor

Note: Baseline was defined as the pre-dose day 1 value. For participants who were on stable ELX/TEZ/IVA (i.e., did not participate in the run-in period), if the pre-dose day 1 value was missing, screening assessments were used as baseline.

Table S14. Post hoc Subgroup Analyses of Proportion of Participants With Sweat Chloride <60 mmol/L Through Week 24.

	**VNZ/TEZ/D-IVA N = 78**
**Genotype Subgroup: *F508del-F508del***	
N1	37
n	36
Proportion, n/N1 (%)	97·3
95% CI for the proportion	(85·8, 99·9)
**Genotype Subgroup: *F508del*-minimal function**	
N1	24
n	21
Proportion, n/N1 (%)	87·5
95% CI for the proportion	(67·6, 97·3)

CI: confidence interval; n: size of subsample; N: total sample size; N1: number of subjects with non-missing sweat chloride at week 16 or week 24; VNZ/TEZ/D-IVA: vanzacaftor/tezacaftor/deutivacaftor

Note: Average through Week 24 is obtained by averaging Weeks 16 and 24.

Table S15. Post hoc Subgroup Analyses of Proportion of Participants With Sweat Chloride <30 mmol/L Through Week 24.

	**VNZ/TEZ/D-IVA N = 78**
**Genotype Subgroup: *F508del-F508del***	
N1	37
n	23
Proportion, n/N1 (%)	62·2
95% CI for the proportion	(44·8, 77·5)
**Genotype Subgroup: *F508del*-minimal function**	
N1	24
n	5
Proportion, n/N1 (%)	20·8
95% CI for the proportion	(7·1, 42·2)

CI: confidence interval; n: size of subsample; N: total sample size; N1: number of subjects with non-missing sweat chloride at week 16 or week 24; VNZ/TEZ/D-IVA: vanzacaftor/tezacaftor/deutivacaftor

Note: Average through Week 24 is obtained by averaging Weeks 16 and 24.

References

1. Quanjer PH, Stanojevic S, Cole TJ, et al. Multi-ethnic reference values for spirometry for the 3-95-yr age range: the global lung function 2012 equations. Eur Respir J. 2012;40(6):1324-43.

2. Miller MR, Hankinson J, Brusasco V, et al. Standardisation of spirometry. Eur Respir J. 2005;26(2):319-38.

3. Counahan R, Chantler C, Ghazali S, Kirkwood B, Rose F, Barratt TM. Estimation of glomerular filtration rate from plasma creatinine concentration in children. Arch Dis Child. 1976;51(11):875-8.

4. Kenward MG, Roger JH. Small sample inference for fixed effects from restricted maximum likelihood. Biometrics. 1997;53(3):983-97.

5. Quittner AL, Modi AC, Wainwright C, et al. Determination of the minimal clinically important difference scores for the Cystic Fibrosis Questionnaire-Revised Respiratory Symptom Scale in two populations of patients with cystic fibrosis and chronic Pseudomonas aeruginosa airway infection. Chest. 2009;135(6):1610-8.

6. Fuchs HJ, Borowitz DS, Christiansen DH, et al. Effect of aerosolized recombinant human DNase on exacerbations of respiratory symptoms and on pulmonary function in patients with cystic fibrosis. N Engl J Med. 1994;331(10):637-42.

7. Ramsey BW, Davies J, McElvaney NG, et al. A CFTR potentiator in patients with cystic fibrosis and the G551D mutation. N Engl J Med. 2011;365(18):1663-72.
